# Investigating PKD2 deficiency-associated cardiomyopathies using hESC-cardiomyocytes and bioengineered 3D ventricular cardiac tissue strips

**DOI:** 10.1038/s41419-026-08639-8

**Published:** 2026-03-25

**Authors:** Jingxuan Li, Wentao Peng, Maxwell Kwok, Huanyu Ding, Duan Zhuo, Bimal Gurung, Ishan Raj Lakhani, Hongyan Yu, Ellen N. Poon, Ronald A. Li, Xiaoqiang Yao

**Affiliations:** 1 School of Biomedical Sciences, Faculty of Medicine, The Chinese University of Hong Kong, Institute of Hematology & Blood Diseases Hospital, Chinese Academy of Medical Sciences & Peking Union Medical College, Hong Kong, China; 2 State Key Laboratory of Experimental Hematology, National Clinical Research Center for Blood Diseases, Haihe Laboratory of Cell Ecosystem, Tianjin, China; 3 Heart and Vascular Institute and Li Ka Shing Institute of Health Science, Faculty of Medicine, The Chinese University of Hong Kong, Hong Kong, China; 4 Southern University of Science and Technology Yantian Hospital, Shenzhen, China; 5 Novoheart, Boston, MA USA; 6 Medera Biopharm, Boston, MA USA

**Keywords:** Embryonic stem cells, Cardiomyopathies

## Abstract

Autosomal dominant polycystic kidney disease is a highly prevalent hereditary renal disorder caused by mutations in either polycystin-1 or polycystin-2. These patients also develop cardiomyopathies. However, the mechanism of how polycystin-2 defects could lead to cardiomyopathies is poorly understood. Moreover, previous studies using animal models cannot fully represent human cardiomyocyte pathophysiology. Human embryonic stem cells were differentiated into cardiomyocytes. These cardiomyocytes were transduced with viral-based polycystin-2-shRNAs, then mixed with an appropriate amount of human fetal fibroblasts, collagen, and Matrigel, and biofabricated into 3D bioengineered ventricular cardiac tissue strips (hvCTS). We used these 3D hvCTS and 2D human embryonic stem cells-derived cardiomyocytes to recapitulate polycystin-2 deficiency-associated cardiac contractile defects and to explore underlying mechanisms. Knockdown of polycystin-2 decreased the contractile force and slowed down the contraction and relaxation velocities in hvCTS, indicative of contractile malfunction. The underlying mechanisms involved an elevated endoplasmic reticulum stress and a decreased activity of sarcoplasmic reticulum Ca^2+^-ATPases. Alleviation of endoplasmic reticulum stress by small molecular chaperones 4-phenylbutyrate/tauroursodeoxycholic acid or stimulation of sarcoplasmic reticulum Ca^2+^-ATPase activity by CDN1163 partially rescued the polycystin-2 deficiency-associated contractile dysfunction in hvCTS. This study used 3D hvCTS and 2D human embryonic stem cells-derived cardiomyocytes as novel disease models to recapitulate PKD2 deficiency-associated contractile defects. We found that knockdown of polycystin-2 induces cardiomyopathies via elevating endoplasmic reticulum stress and decreasing sarcoplasmic reticulum Ca^2+^-ATPase activity. The results provide novel insights about polycystin-2 deficiency-associated cardiomyopathies in polycystic kidney disease patients.

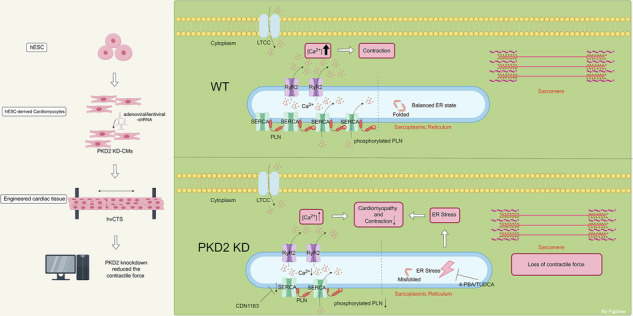

## Introduction

Autosomal dominant polycystic kidney disease (ADPKD) is a common hereditary disorder caused by loss-of-function heterozygous mutations either in polycystin-1 (PKD1) or polycystin-2 (PKD2) [1]. In addition to kidney disease, ADPKD patients develop cardiac diseases, with more than 90% of the patients exhibiting left ventricular hypertrophy at death, 18–27% having cardiac valvular abnormality, and ~26% having arrhythmia [2, 3]. Evidence suggests that the cardiovascular diseases in ADPKD patients can either result indirectly from renal dysfunction or result directly from PKD1/PKD2 defects in cardiomyocytes [3–6].

Ca^2+^ signaling plays an important role in myocardial functions. During a heartbeat, an action potential activates L-type Ca^2+^ channels (LTCC) to induce extracellular Ca^2+^ influx in cardiomyocytes. This, in turn, activates type 2 ryanodine receptors (RyR2) to cause a large amount of Ca^2+^ release from sarcoplasmic reticulum (SR), resulting in cytosolic Ca^2+^ rise and consequent cardiomyocyte contraction [7]. The magnitude of cytosolic Ca^2+^ transient is positively correlated with SR Ca^2+^ content, which in turn is regulated by RyR2 and sarcoplasmic reticulum Ca^2+^-ATPases (SERCA) [8]. In addition to its role in cardiomyocyte contraction, Ca^2+^ signaling also regulates other processes, such as autophagy, endoplasmic reticulum (ER) stress, and myocardial cell death [7, 9]. Dysregulation in Ca^2+^ signaling may cause cardiomyopathies [7, 9]. For example, depletion of SR Ca^2+^ content results in an unfavorable SR microenvironment to disrupt protein folding, causing accumulation of unfolded or misfolded proteins to elevate ER stress, resultant in cell death and cardiomyopathy [9–11].

PKD2 is a Ca^2+^-permeable nonselective cation channel predominantly localized in ER/SR. In animal models, PKD2 deficiency displays cardiac septal defects [4], decreases cardiac contraction [12, 13], and increases the incidence of cardiac desynchrony [14]. However, the mechanism by which PKD2 deficiency can lead to cardiac disorders is poorly understood. One possibility is that PKD2 deficiency may alter Ca^2+^ signaling, resulting in cardiomyopathies [13–17]. Previously, we and others have reported that PKD2 deficiency may reduce RyR2-mediated SR Ca^2+^ release, consequently impairing autophagy to induce apoptosis [15, 16]. Note that the amount of SR Ca^2+^ release is positively correlated with SR Ca^2+^ content, the depletion of which may elevate ER stress. Therefore, it is reasonable to speculate that PKD2 deficiency may dysregulate SR Ca^2+^ content, consequently elevating ER stress as another culprit for cardiomyopathy. However, up to the present, the role of PKD2 deficiency on the ER stress of cardiomyocytes remains unclear. Furthermore, although several studies have investigated the effect of PKD2 on SERCA expression in mouse cardiomyocytes [12], the molecular mechanism of SERCA involvement in PKD deficiency-induced cardiomyopathy is incompletely understood.

Note that almost all previous studies used cultured rodent cells or PKD2 knockout mice/zebra fish model to investigate the role of PKD2 in cardiomyopathies. However, this is problematic because there are considerable differences in electrophysiological properties and Ca^2+^ handling mechanisms between human and animal cardiomyocytes [18, 19]. In this regard, human embryonic stem cell-derived cardiomyocytes (hESC-CMs) offer great advantages over rodent cardiomyocytes, because hESC-CMs are physiologically more similar to human myocardium [18–20]. An even more attractive option is to bioengineer 3D cardiac strips from 2D cultured hESC-CMs [21]. The bioengineered 3D cardiac strips have multicellular organization and a 3D structure that can better mimic the in vivo heart [21, 22]. The 3D microenvironment also provides a necessary niche to promote maturation of hESC-CMs, facilitate cell alignment, and enhance force generation [21–23].

In the present study, hESC-CMs were biofabricated into 3D human ventricular cardiac tissue strips (hvCTS). These 3D hvCTS and 2D cultured hESC-CMs were used as novel disease models to investigate PKD2 deficiency-associated contractile dysfunction and possible underlying mechanisms related to ER stress and SERCA.

## Methods

### Cardiomyocyte differentiation

The methods for differentiation of H7-hESCs into H7-cardiomyocytes (H7-CMs) were as described previously [24, 25]. Briefly, H7-hESCs were maintained in E8 media (Thermo Fisher, USA) under 5% CO_2_ in a humidified incubator at 37 °C. Differentiation was induced using a monolayer system in RPMI/B27 medium lacking insulin (Thermo Fisher, USA) by the addition of glycogen synthase kinase 3 inhibitor CHIR99021 (6 μM; Selleckchem, Houston, USA) from days 0 to 2, followed by the addition of Wnt inhibitor IWR-1 (10 μM; Sigma-Aldrich, NY, USA) from days 3 to 5. H7-CMs were maintained in RPMI/B27 medium containing insulin from day 7 (Thermo Fisher, USA). Beating was usually observed on days 7 and 8. At around day 14–16, the cells were dissociated with trypsin in DPBS (Gibco, NY, USA) at 37 °C for 5 min, followed by spinning at 1000 rpm for 5 min and then resuspended in RPMI/B27+ with Y27632 (1 μl/ml). These cells were ready for further experiments. Flow cytometry analysis of the differentiated H7-CMs showed that ~95% of cells were cTnT-positive cardiomyocytes. cTnT immunostaining of the cells showed clear, organized sarcomeres, also indicative of cardiomyocytes (Fig. S1).

The methods for differentiation of HES2-hESCs into HES2-cardiomyocytes (HES2-CMs) were as described elsewhere [25, 26]. Briefly, HES2 cells from passage 40–80 were suspension cultured to form embryoid bodies, and then differentiated to cardiomyocytes in culture media with addition of recombinant human bone morphogenetic protein 4 (BMP-4) and Rock inhibitor Y27632 (1 μl/ml) at day 0, followed by BMP-4, ascorbic acid (AA, Sigma-Aldrich, NY) and activin-A at day 1, and then AA and IWR-1 at day 4 under a hypoxic condition of 5% O_2_. At 8 days after cardiac differentiation, the embryoid bodies were transferred to a normoxic environment and maintained in StemPro34 SFM (Invitrogen, NY, USA) with AA medium for further characterization. HES2-CMs were isolated by digesting beating cardiospheres (day 15) with Trypsin (1 mg/ml, Gibco, NY, USA) and EDTA (ethylenediaminetetraacetic acid, 0.05%, Life Technologies) at 37 °C for 15–20 min, followed by spinning at 1000 rpm for 5 min and then resuspended in RPMI/B27+ with AA and Y27632. These cells were ready for further experiments.

### Adenoviral- and lentiviral-based short hairpin RNA

H7-CMs were transduced with adenoviral-based PKD2 short hairpin RNA (ad-PKD2-shRNA1) using the AdEasy system (Agilent Tech, USA). The sequence of PKD2-shRNA used was 5’-CCAGGACTTGAGAGATGAAAT-3’. The shRNA against human PKD2 was synthesized, annealed, and subcloned into pAdTrack-U6. Adenoviral recombinants were generated in *Escherichia coli* strain BJ5183 with an adenoviral backbone plasmid, pAdEasy-1. Positive recombinants were linearized by PacI digestion and transfected into HEK-293A cells for virus packaging. The medium and cells were collected until the cytopathic effect was apparent. Recombinant adenoviruses were purified by AdenoPACK 20 Maxispin column (Satorius, Germany) and concentrated by VIVA-SPIN 20 concentrator (100 kDa cut-off, Satorius, Germany). Scrambled shRNA in an adenoviral vector (Ad-SCR-shRNA) was used as a control. H7-CMs were transduced with adenoviral-based constructs. After 48–72 h, the cells were ready for experiments. All subsequent experiments were performed when the cell confluence was about 70%.

HES2-CMs were transduced with lentiviral-based PKD2 shRNA (lenti-PKD2-shRNA2). The sequence of PKD2-shRNA2 was 5’-GCAGAGATTGAGGAAGCTAAT-3’. The PKD2-shRNA2 sequence was cloned into the GV298 vector. Lentiviral packaging plasmid psPAX2 and envelope plasmid pMD2.G were obtained from AddGene. Lentiviruses were obtained with a commercial lentivirus package kit following the manufacturer's instructions (Thermo Fisher, USA).

### Crispr/Cas9-based homozygous PKD2 gene knockout

A CRISPR/Cas9-mediated gene knockout was generated in the human H1 cell line through a commercial genome-editing service (Ubigene, Guangzhou, China). Two pairs of guide RNAs (gRNAs) targeting the genomic region of interest were designed and validated for on-target efficiency. Pair 1 consisted of gRNA1 (forward orientation sequence: 5′-CTGCTGAGGCTGCACGCGACTGG-3′; predicted score: 92, 0.76) and gRNA2 (reverse orientation sequence: 5′-CTCTCCGCCCAGGCCACTCGGGG-3′; predicted score: 86, 0.75), which were intended to induce a ~542 bp deletion. Pair 2 consisted of gRNA3 (forward orientation sequence: 5′-CTTGGGGGCTACCACGGCGCGGG-3′; predicted score: 93, 0.83) and gRNA4 (forward orientation sequence: 5′-CGAGATGCAGCGCATCCGGCAGG-3′; predicted score: 92, 0.71), designed to generate a ~259 bp deletion. gRNA oligos were cloned into the CRISPR/Cas9 expression vector and transfected into H1 cells following the manufacturer’s optimized protocol. Edited cells were enriched and clonally expanded, and genomic DNA from single-cell–derived colonies was subjected to PCR and Sanger sequencing to confirm the expected deletion events and establish validated knockout clones.

### Fabrication of human ventricular cardiac tissue strips (hvCTS) and force measurement

The methods were as described previously with slight modification [21, 27]. Briefly, after adenoviral/lentiviral-shRNA transduction, 8–15 million cTnT^+^ H7-CMs or HES2-CM2 were resuspended in B27+/RPMI 1640 with ascorbic acid and Y27632, and then incubated in 37 °C CO_2_ incubator for 48 h to allow small clusters to form. The CM clusters were recollected by centrifugation at 1000 rpm for 5 min. For each strip, 1 × 10^6^ CMs were mixed with 0.1 × 10^6^ human fetal fibroblasts. The cell mixture was combined with ice-cold bovine collagen type I (Sigma-Aldrich, NY, USA) and Matrigel (BD Biosciences, San Jose, USA) at a ratio of 1:8:1 (v/v/v). The cells/ collagen/Matrigel mixture was then pipetted into a custom mold made of polydimethylsiloxane (PDMS) elastomer, filling the rectangular well with 110 μl (10^6^ cells/ well), and then incubated at 37 °C, 5% CO_2_ for 1 h to allow the collagen to polymerize. They were then bathed with differentiation medium (StemPro34, ascorbic acid, and GlutaMAX-I) and maintained in culture with daily half-medium exchanges. Inserts in the casting mold were removed at 48 h, yielding a self-assembled hvCTS held between 0.5-mm-diameter PDMS posts at each end. At day 5 after hvCTS creation, culture medium was changed to high-glucose Dulbecco’s modified Eagle medium (DMEM) supplemented with 10% newborn bovine serum (Atlanta Biologicals, Lawrenceville, USA), 1% penicillin/streptomycin (Gibco, Carlsbad, USA), and 0.2% amphotericin B (Sigma-Aldrich, NY). Force measurement was taken at day 7 for H7-CMs-derived hvCTS and day 11 for HES2-CMs-derived hvCTS.

For force measurement, the integrated flexible PDMS posts were used as force sensors, with the post-deflection captured in real time with a high-speed camera (100 frames/s) and LabView software (National Instruments, Austin, USA), applying a beam-bending equation from elasticity theory to calculate twitch force, as described previously [21, 27]. These measurements were obtained with the hvCTS maintained in the original molds where they were created, and all experiments were carried out under sterile conditions inside a laminar flow hood, with the hvCTS bathed in culture medium at 37 °C, with and without electrical field stimulation. Force measurement data were analyzed by the software “CTS_Processor” and “MyHeart_DataAnalyzer”. Note that preparation of hvCTS involved multiple steps with complicated procedures that lasted 7–11 days. Batch-to-batch variety in the hvCTS contractile force was inevitable. Therefore, it is essential to have good controls for each experiment series. For that reason, in each experiment we have 6–9 control hvCTS and comparable numbers of treatment hvCTS (such as PKD2 knocknown, etc).

When necessary, electrical pacing was provided by the AMPI Master-9 Programmable Pulse Stimulator (Global Biotech, USA). Pacing frequencies were adjusted accordingly on the Master-9 system to allow for electrical stimulation of force generation.

### RNA purification and quantitative real-time PCR

Total RNA was extracted from H7-CMs using TRIzol, according to the manufacturer’s instructions (Invitrogen, USA). The quality of the RNA was determined by a Nanodrop 2000 (Thermo Fisher, USA). RNA was subjected to reverse transcription using a first-strand cDNA synthesis kit (TRANSGEN, China). Samples are analyzed using SYBR Green qPCR SuperMix (Takara, Japan). The qRT-PCR was performed using the ViiATM7 real-time PCR system (Applied Biosystems, Waltham, USA). The level of mRNA was quantified using the ΔΔCt method with GAPDH as the housekeeping gene for internal normalization.

### Western blots and co-immunoprecipitation

For western blot experiments, H7-CM or HES2-CM lysates prepared in RIPA lysis buffer were resolved on SDS/PAGE gel and were blotted onto a polyvinylidene difluoride (PVDF) membrane. The protein concentration was measured by using the DC protein assay (Bio-Rad, USA). The PVDF membrane was blocked with 5% BSA in TBS for 1 h, followed by incubation with primary antibodies (1:500–1:2000 dilutions) overnight at 4 °C, followed by the appropriate horseradish peroxidase-conjugated secondary antibody (1:1000 dilution). Immunodetection was accomplished using horseradish peroxidase-conjugated secondary antibody and the enhanced chemiluminescence detection system. Primary antibodies were PKD2 (19126-1-AP, Proteintech, USA), SERCA (9580P, Cell Signaling Technology, USA), GRP78 (11587-1-AP, Proteintech, USA), ATF4 (10835-1-AP, Proteintech, USA), Cleaved ATF6 (24169-1-AP, Proteintech, USA), CHOP (15204-1-AP, Proteintech, USA), Spliced XBP1 (25997-1-AP, Proteintech, USA) and β-tubulin (2146, Cell Signaling Technology, USA).

For coimmunoprecipitation, 200 μg of HES2-CMs lysate proteins were immunoprecipitated with 2 μg of anti-PKD2 antibody or anti-PLN antibody with protein A Agarose beads (Beyotime, China) at 4 °C. Negative controls were rabbit preimmune IgG (Proteintech, USA).

### Immunostaining of the hvCTS section

hvCTS were fixed with 4% paraformaldehyde in phosphate-buffered solution. 5 mm-thick frozen sections were prepared. Endogenous peroxidase was blocked with 3% H_2_O_2_/methanol for 12 min, incubated with primary anti-cTnT or anti-α-actinin antibody (1:200 dilution) overnight at 4 °C, followed by fluorescent secondary antibody at 37 °C for 1 h in the dark. The slides were mounted with DAPI-containing glycerol (ZLI-9557, ZSBIO). Pictures were taken under a Leica TCS SP8 inverted confocal microscope.

### Cardiomyocyte hypertrophy

Cardiomyocyte hypertrophy in H7-CMs was measured as described previously [25]. Briefly, H7-CMs were transduced with or without adenoviral-based PKD2-shRNA constructs. 4 days after transduction, the cardiomyocytes were visualized with an Olympus inverted microscope equipped with a Polaroid digital camera under 320 magnification, and the area of 100 randomly selected cells was determined using Image J software.

### Flow cytometry-based cell necrosis and apoptosis

Necrosis and apoptosis of H7-CMs were determined by flow cytometric analysis. Propidium iodide (PI) was used to detect necrotic cells, while phospholipid binding proteins Annexin V was used to detect apoptotic cells. Briefly, cells were incubated with APC-Annexin V (550474, BD, USA) or propidium iodide—PI (ab14083, Abcam, USA) for 30 min in the dark. Flow cytometry was performed by BD FACSymphony A5.2 SORP Flow Cell Analyzer and BD FlowJo software.

### TUNEL assay with cTnT staining

TUNEL staining of H7-CMs was conducted using the BeyoClick™ EdUTP TUNEL Apoptosis Assay Kit with AF647 (C1179). Briefly, the cells were fixed with 4% paraformaldehyde for 30 min, followed by EdUTP labeling in a humidified chamber at 37 °C for 1 h and then EdUTP assay at room temperature for 30 min in the dark according to the manufacturer’s instructions. Cells were incubated with primary anti-cTnT antibody (1:500 dilution), overnight at 4 °C, followed by fluorescent secondary antibody at 37 °C for 1 h in the dark. The cell nuclei were further stained with 1 μg/ml 4,6 diamidino-2-phenylindole (DAPI) for 30 min in the dark at room temperature. The fluorescence was observed and photographed under a Leica TCS SP8 inverted confocal microscope with excitation wavelengths in the range of 488 nm and analyzed using ImageJ software.

### Measurements of cytosolic Ca^2+^ and store Ca^2+^ content

Cytosolic Ca^2+^ [Ca^2+^]_i_ in H7-CMs was measured as described previously [16]. Briefly, H7-CMs were loaded with 5 µM Fluo-4/AM (Thermo Fisher, USA) for 30 min or 5 µM Calbryte 590 for 1 h with 0.02% pluronic F127 in the dark at 37 °C. Cytosolic Ca^2+^ oscillations were measured in Tyrode’s solution containing in mM: NaCl 140, KCl 5.4, CaCl_2_ 1.8, MgCl_2_ 1, glucose 10, HEPES 10, adjusted to pH 7.40 with NaOH. Fluo-4 was excited at the 488 nm line and captured at wavelengths 505–530 nm, while Calbryte 590 was excited at 559 nm and captured at wavelengths of 575–620 nm. Data acquisition was performed using a confocal microscope (Olympus FV1000). The amplitude of cytosolic Ca^2+^ oscillations was displayed as a ratio of maximal fluorescence increase relative to the basal intensity (*F*1/*F*0). *T*_1/2_ is defined as the time needed for [Ca^2+^]_*i*_ dropping to 50% of its peak value.

SR Ca^2+^ content was monitored by loading H7-CMs with 10 µM Mag-fluo-4/AM (F14201, Thermo Fisher, USA) and 0.02% pluronic F127 for 30 min in the dark at 37 °C. Measurement conditions were the same as in the cytosolic Ca^2+^ measurement.

### Statistical analysis

All experimental results are expressed as mean ± standard deviation (mean ± SD) or mean ± standard error of mean (mean ± SEM). The data between the two groups were compared using a *t*-test. The comparison between multiple groups was performed using one-way ANOVA with Newman–Keuls post hoc test. Comparison between two plotted curves was performed using two-way ANOVA followed by Bonferroni post-test. *P* < 0.05 was considered statistically different between groups. Each experiment was repeated at least three times. GraphPad Prism 8 software was used for statistics and graphing.

### Ethics approval

The use of human embryonic stem cell lines was approved by the University Safety Office, The Chinese University of Hong Kong. The approved project “Using 2D and 3D models of engineering human cardiac tissues to investigate PKD2-associated cardiomyopathies” with reference number 14110320. The experiments were conducted in compliance with the guidelines and regulations of the International Society for Stem Cell Research (ISSCR). No experiments were performed with live vertebrates.

## Results

### PKD2 knockdown reduced the contractile force of spontaneous and pacing-inducing contraction in 3D hvCTS

**Fig. 1:**
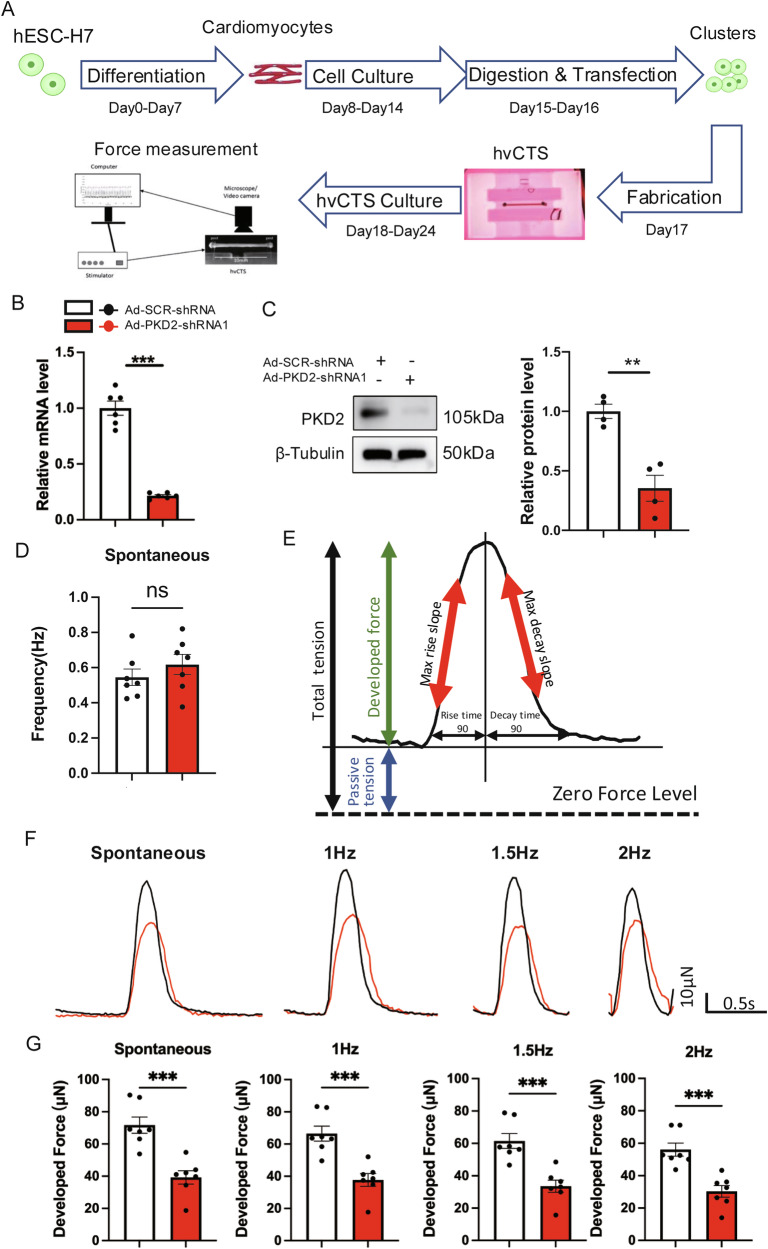
PKD2 knockdown reduced the contractile force of 3D H7-CMs-derived hvCTS. **A** Schematic diagram of hvCTS fabrication and contractile force measurement. **B** and **C** Ad-PKD2-shRNA1 knocked down the expression of PKD2 at the mRNA level (**A**, *n* = 6) and protein level (**B**, *n* = 4). In **B**, western blot images (left) and summary data normalized to β-tubulin (right). **D** Spontaneous contraction frequency of hvCTS. *n* = 7. (**E**) Schematic illustration of parameters used for twitch analysis. **F** and **G** Representative contractile traces of hvCTS (**F**) and data summary (**G**) showing that PKD2 knockdown with Ad-PKD2-shRNA1 reduced the developed force at different electrical pacing frequencies. *n* = 7. Mean ± SEM. ns not significant; *P < 0.05; **P < 0.01; ***P < 0.001.

H7-CMs were bioengineered to make 3D hvCTS (Fig. 1A). The hvCTS is approximately 10 mm long and 1 mm thick (Fig. 1A), which can produce an average maximum twitch stress of 660 μN/mm^2^ at *L*_max_ [21], which is close to the value in neonatal myocardium. An Ad-PKD2-shRNA1 was constructed, which could knockdown PKD2 mRNA level by 79% and PKD2 protein level by 65%, respectively (Fig. 1B and C). Spontaneous and electrical pacing (1–2 Hz)-induced contractions of hvCTS were recorded. Knockdown of PKD2 did not alter the frequency of spontaneous contraction (Fig. 1D). Figure 1E illustrates the meaning of different contractile parameters measured from hvCTS, including developed force, passive tension, rise time 90, maximal rise slope, decay time 90, and maximal decay slope.

**Fig. 2:**
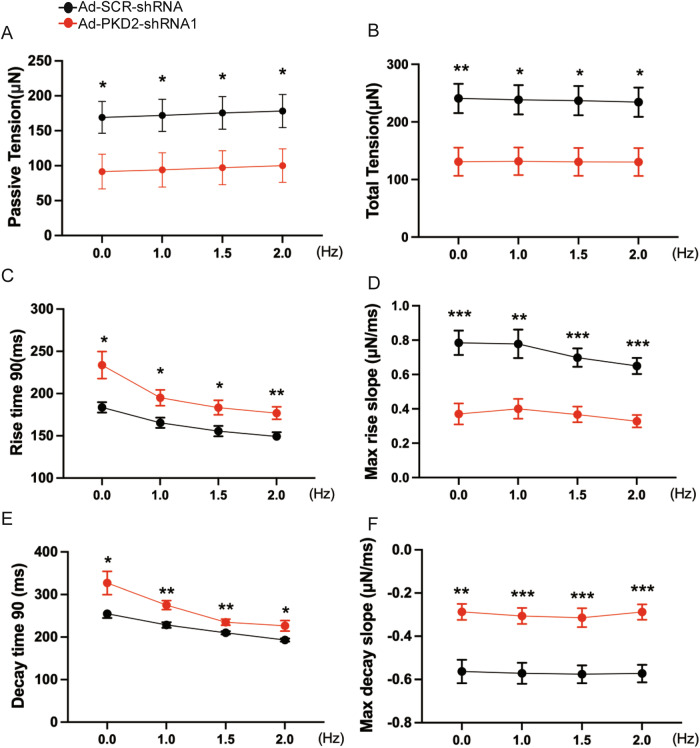
PKD2 knockdown slowed down the rise time kinetics and decay time kinetics of developed force. Data summary showing that PKD2 knockdown with Ad-PKD2-shRNA1 reduced the passive tension (**A**) and total tension (**B**) at different electrical pacing frequencies. Data summary showing that PKD2 knockdown with Ad-PKD2-shRNA1 prolonged the rise time 90 (**C**) and decay time 90 (**D**), and decreased the maximal rise slope (**E**) and maximal decay slope (**F**) at different pacing frequencies in H7-CMs-derived hvCTS. *n* = 7. Mean ± SEM. *P < 0.05; **P < 0.01; ***P < 0.001.

We examined the effect of PKD2 knockdown on developed force and passive tension. The passive tension is the baseline force generated by hvCTS between two force-sensing cantilever posts, whereas the developed force is the total force minus the passive tension. PKD2 knockdown reduced the developed force (Fig. 1F and G), passive tension (Fig. 2A), and total tension (Fig. 2B) of spontaneous contractions and pacing (1–2 Hz)-induced contractions. As pacing frequency increased, there was a trend of slight decrease in developed force for hvCTS (Fig. 1F and G), which is a characteristic of relatively immature newborn myocardium [20].

Cardiomyocytes differentiated from different hESCs and/or using different differentiation protocols exhibit great phenotypic heterogeneity [28, 29]. Therefore, we generated human cardiomyocytes using another hESC line, HES2, and using a different differentiation method based on suspended embryonic bodies (Fig. S2) [26]. Furthermore, to avoid shRNA off-target effect, we used a lentiviral-carried PKD2-shRNA (lenti-PKD2-shRNA2) that targeted PKD2 at a different site from that of Ad-PKD2-shRNA1. Lenti-PKD2-shRNA2 knocked down the PKD2 protein expression by 60% (Fig. S3A). 3D hvCTS were made from these transduced HES2-CMs. The results showed PKD2 knockdown did not alter the frequency of spontaneous contraction (Fig. S3B), but markedly reduced the developed force (Fig. S3C and D), passive tension, and total tension (Fig. S3E–G) of spontaneous and pacing-induced contractions in HES2-CMs-derived hvCTS.

hvCTS became unstable under high-frequency electrical pacing, with variability in developed force reaching more than 5% and 10% at 2.5 and 3 Hz pacing frequency, respectively (Fig. S4). Therefore, only the data from the pacing frequency ≤2 Hz were used for further analysis.

### PKD2 knockdown slowed down the rise time kinetics and decay time kinetics of developed force in 3D hvCTS

We further investigated the effect of PKD2 knockdown on the rise time kinetics and decay time kinetics of the developed force in H7-CMs-derived hvCTS. As shown in Fig. 2C and D, PKD2 knockdown with Ad-PKD2-shRNA1 prolonged the rise time 90 and decreased the maximal rise slope of developed force for spontaneous and pacing-induced contractions, indicating that PKD2 knockdown slowed down the contraction velocity. Furthermore, PKD2 knockdown prolonged the decay time 90 and decreased the maximal decay slope of developed force for spontaneous and pacing-induced contractions, indicating that PKD2 knockdown also slowed down the relaxation velocity (Fig. 2E and F).

Similarly, in HES2-CMs-derived hvCTS, knockdown of PKD2 with lenti-PKD2-shRNA2 prolonged the rise time (Fig. S5A and B) and decay time kinetics (Fig. S5C and D) of developed force.

### PKD2 deficiency elevated ER stress level, promoted apoptosis and induced cardiomyocyte hypertrophy

**Fig. 3:**
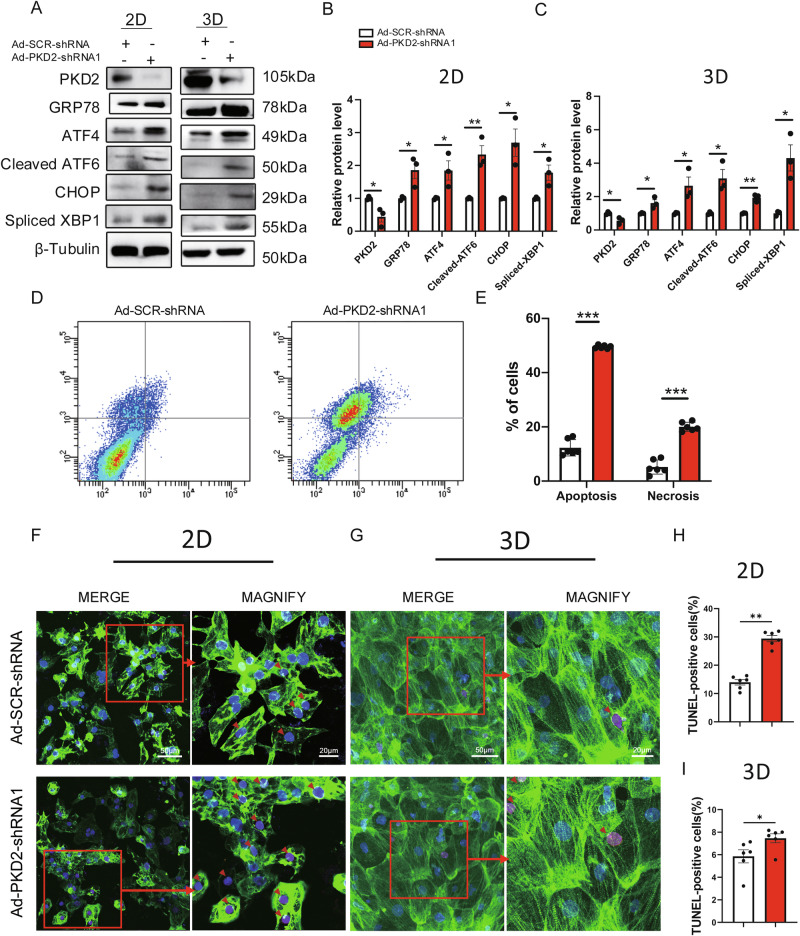
PKD2 knockdown elevated ER stress levels and promoted apoptosis. **A–C** PKD2 knockdown with Ad-PKD2-shRNA1 increased the expression of ER stress markers, including GRP78, ATF4, cleaved ATF6, CHOP, and spliced XBP1. Shown are representative western blot images (**A**) and data summary (**B** for H7-CMs, **C** for hvCTS). *n* = 3. PKD2 knockdown promoted apoptosis and necrosis of H7-CMs, as determined by flow cytometer analysis. Shown are representative images (**D**) and data summary (**E**). *n* = 6. PKD2 knockdown promoted apoptosis of H7-CMs, as determined by TUNEL assay. Shown are representative pictures (**F**) and summary data (**H**) of TUNEL-positive cells. PKD2 knockdown promoted cardiomyocyte apoptosis in hvCTS, as determined by TUNEL assay. Shown are representative pictures (**G**) and summary data (**I**) of TUNEL-positive cells. Violet, TUNEL; Green, cardiomyocyte marker cTnT; Blue, DAPI staining. In magnified images, small red arrows point to the TUNEL-positive cells. *n* = 6. Mean ± SEM. *P < 0.05; **P < 0.01; ***P < 0.001.

Western blot was used to determine the expression levels of ER stress-related proteins, including glucose-regulated protein 78 (GRP78), activating transcription factor 4 (ATF4), cleaved activating transcription factor 6 (cleaved ATF6), C/EBP homologous protein (CHOP) and spliced X-box binding protein 1 (spliced XBP1). PKD2 knockdown with Ad-PKD2-shRNA1 increased the expression levels of all these markers in both 2D cultured H7-CMs and 3D hvCTS (Fig. 3A–C). Flow cytometry results showed that PKD2 knockdown promoted apoptosis and necrosis in H7-CMs, as indicated by increases in annexin V-positive cells and propidium iodide (PI)-positive cells, respectively (Fig. 3D and E). TUNEL assay confirmed that PKD2 knockdown promoted apoptosis of 2D H7-CMs (Fig. 3F and H) and 3D hvCTS (Fig. 3G and I).

PKD2 knockdown with Ad-PKD2-shRNA1 also increased the expression of cardiac hypertrophic markers atrial natriuretic factor (ANF) and ACTA1 at the mRNA level (Fig. S6A) and caused a cell size enlargement of H7-CMs (Fig. S6B and C).

### Small molecular chaperones partially rescued the contractile deficiency of PKD2 knockout 3D hvCTS

**Fig. 4:**
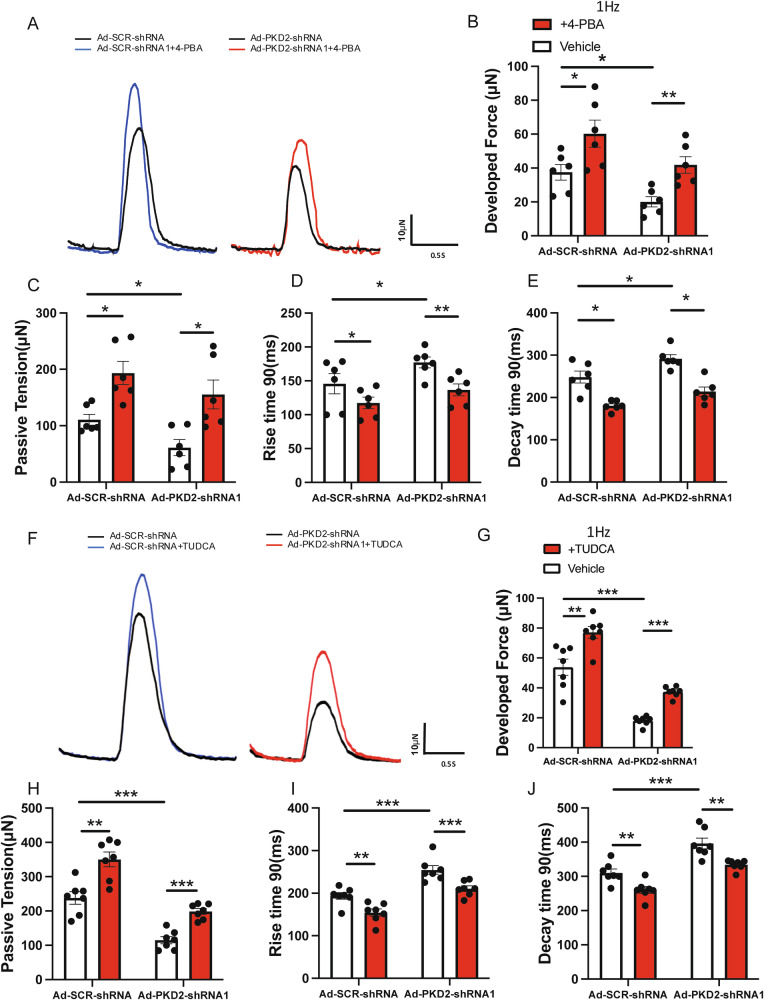
Small molecular chaperones partially rescued the PKD2 deficiency-associated contractile deficiency. **A** Representative contractile trace of hvCTS under 1 Hz electric pacing before (black trace) and after (red or blue trace) 3 days’ treatment with 5 mM 4-PBA. Right, PKD2 knockdown; Left, control with Ad-SCR-shRNA. Data summary showing that 4-PBA treatment improved the contractile function in hvCTS samples with or without PKD2 knockdown, regarding developed force (**B**), passive tension (**C**), rise time 90 (**D**), and decay time 90 (**E**). *n* = 7. **F** Representative contractile traces of hvCTS under 1 Hz electric pacing before (black trace) and after (red or blue trace) 3 days’ treatment with 100 μM TUDCA. Right, PKD2 knockdown; Left, control with Ad-SCR-shRNA. **G–J** Data summary showing that TUDCA treatment improved the contractile function in hvCTS with or without PKD2 knockdown. *n* = 6. Mean ± SEM. *P < 0.05; **P < 0.01; ***P < 0.001.

Small molecular chaperones 4-phenylbutyrate (4-PBA) and tauroursodeoxycholic acid (TUDCA) can improve ER protein folding, thereby alleviating ER stress [30, 31]. 4-PBA and TUDCA treatment increased the developed force and passive tension in both PKD2 knockdown hvCTS and control hvCTS (Fig. 4A–C and F–H). 4-PBA and TUDCA also decreased the rise time 90 and decay time 90 in both groups (Fig. 4D, E, I and J). Interestingly, 4-PBA and TUDCA could improve the developed force by a greater percentage in PKD knockdown hvCTS than in control hvCTS (4-PBA: 61 ± 7% in control vs. 119 ± 21% in PKD2 knockdown, *p* < 0.05; TUDCA: 54 ± 21% in control vs. 113 ± 11% in PKD2 knockdown, *p* < 0.05).

### PKD2 knockdown decreased the magnitude of cytosolic Ca^2+^ oscillations and SR Ca^2+^ oscillations in response to 1 Hz electric pacing

**Fig. 5:**
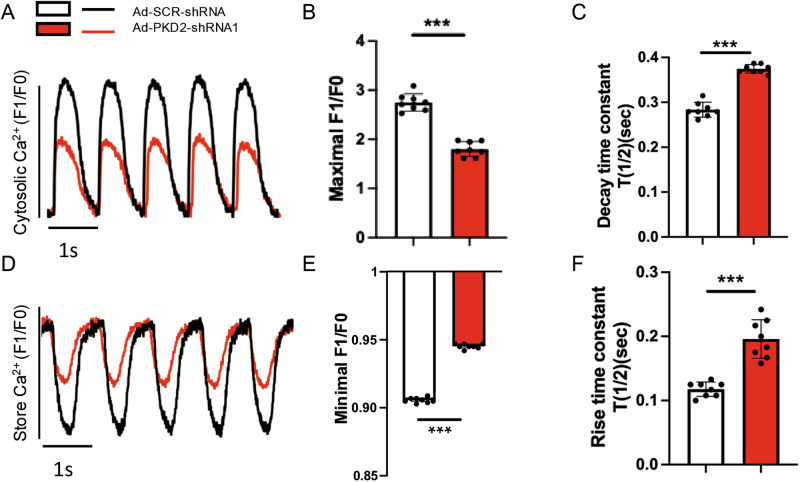
PKD2 knockdown decreased the magnitude of cytosolic and SR Ca^2+^ oscillations. Knockdown of PKD2 with Ad-PKD2-shRNA1 reduced the magnitude of cytosolic Ca^2+^ oscillations and slowed down decay time kinetics in H7-CMs under 1 Hz electrical pacing. Shown are representative traces of cytosolic Ca^2+^ oscillations (**A**) and data summary of maximal amplitude value (**B**) and decay time constant *T*_1/2_ (**C**). *T*_1/2_ represents the time when the Ca^2+^ amplitude decays to 50% of its peak value. *n* = 8. **D–F** Knockdown of PKD2 with Ad-PKD2-shRNA1 decreased the magnitude of SR Ca^2+^ oscillations and slowed down the rise time constant T_1/2_ in H7-CMs under 1 Hz electrical pacing. Shown are representative traces of oscillatory SR Ca^2+^ reduction (**D**) and data summary of minimal SR Ca^2+^ (**E**) and rise time constant T_1/2_ for the recovery/rising phase of SR Ca^2+^ (**F**). *n* = 8. Mean ± SEM. ***P < 0.001.

We examined the effect of PKD2 knockdown on electrical pacing-induced cytosolic Ca^2+^ oscillations in 2D cultured H7-CMs. Knockdown of PKD2 with Ad-PKD2-shRNA1 reduced the amplitude of cytosolic Ca^2+^ oscillation (Fig. 5A and B) and slowed down the decay phase of cytosolic Ca^2+^ oscillations, as indicated by an increased decay time *T*_1/2_ (Fig. 5A and C), suggesting that PKD2 knockdown decreased the SERCA activity and SR Ca^2+^ content.

A low-affinity fluorescence Ca^2+^ dye Mag-fluo-4 was used to monitor SR Ca^2+^ change. Electrical pacing induced oscillatory SR Ca^2+^ decrease (Fig. 5D and E). PKD2 knockdown reduced the magnitude of SR Ca^2+^ oscillations (Fig. 5D and E) and slowed down the rise time *T*_1/2_ of SR Ca^2+^ oscillations (Fig. 5D and F), again supporting that PKD2 knockdown decreased the SERCA activity and SR Ca^2+^ content.

Treatment of H7-CMs with an LTCC inhibitor, nifedipine, or a RYR2 inhibitor each markedly reduced the magnitude of electrical pacing-induced cytosolic Ca^2+^ oscillations. After nifedipine or ryanodine treatment, the residual Ca^2+^ oscillations became very small, and there was essentially no difference between the control and PKD2 knockdown groups (Fig. S7), suggesting that LTCC and RYR2 also contribute to the PKD2 deficiency-induced Ca^2+^ abnormality.

### PKD2 deficiency decreased the expression and activity of SERCA in H7-CMs

**Fig. 6:**
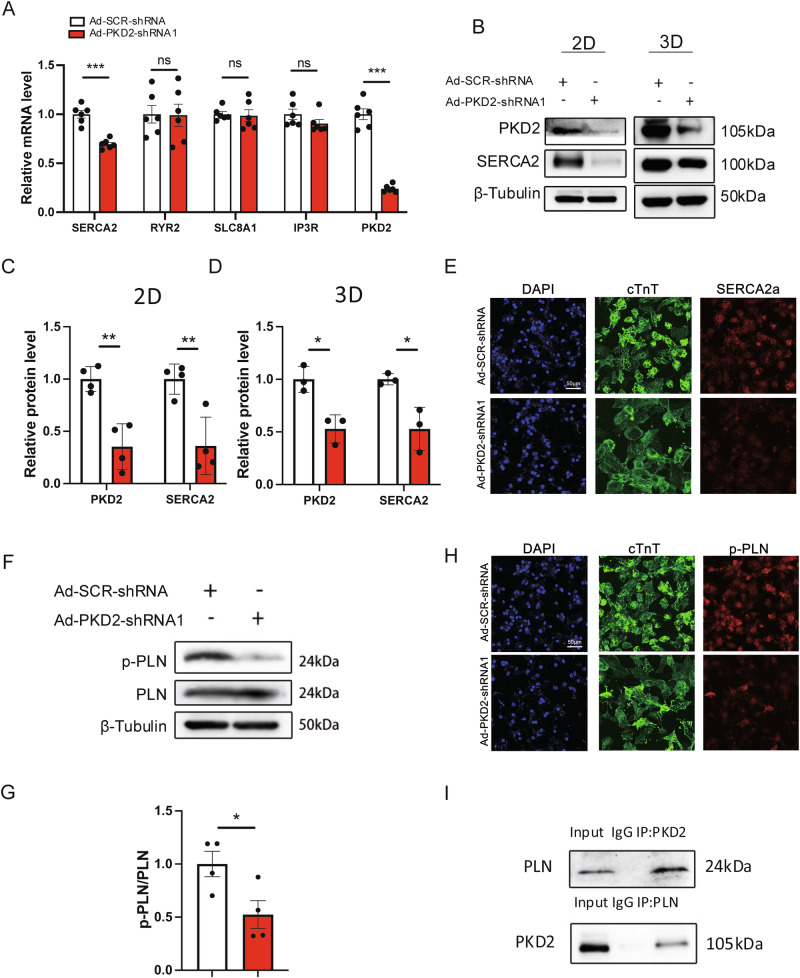
PKD2 knockdown decreased the expression of SERCA2 and phosphorylated PLN. **A** PKD2 knockdown decreased the expression of SERCA2 mRNA by qPCR in H7-CMs. *n* = 6. PKD2 knockdown decreased the expression of SERCA2 proteins in H7-CMs (*n* = 4) and H7-CMs-derived hvCTS (*n* = 3). Shown are representative western blot images (**B**) and data summary (**C** and **D**). **E** PKD2 knockdown reduced the expression of SERCA in H7-CMs by cellular immunostaining. Shown are representative pictures of H7-CMs co-stained with anti-SERCA2a antibody (Red) and anti-cTnT antibody (Green). Blue, DAPI. *n* = 3. PKD2 knockdown with Ad-PKD2-shRNA1 reduced the PLN phosphorylation. *n* = 4. Shown are representative western blot images (**F**) and data summary (**G**). **H** PKD2 knockdown reduced the expression of phosphorylated PLN in H7-CMs by cellular immunostaining. Shown are representative immunostaining pictures of H7-CMs co-stained with anti-pPLN antibody (red) and anti-cTnT antibody (Green). Blue, DAPI. *n* = 3. **I** PKD2 and PLN could pull down each other. *n* = 4. Mean ± SEM. ns not significant; *P < 0.05; **P < 0.01; ***P < 0.001.

The effect of PKD2 knockdown on the mRNA expression levels of a panel of Ca^2+^-handling proteins was examined in 2D cultured H7-CMs. PKD2 knockdown decreased the mRNA level of SERCA2, but did not alter the mRNA level of RYR2, IP_3_R, and SLC8A1 (Na^+^–Ca^2+^ exchanger) (Fig. 6A). Western blot experiments validated that PKD2 knockdown suppressed the expression of SERCA2 in 2D cultured hESC-CMs and 3D hvCTS (Fig. 6B–D), which was further confirmed by cellular fluorescent immunostaining (Fig. 6E).

Phospholamban (PLN) is a protein that can regulate SERCA activity via PLN phosphorylation [32]. Western blot analysis showed that PKD2 knockdown reduced the expression level of phosphorylated PLN (Fig. 6F and G), which was confirmed by cellular fluorescent immunostaining (Fig. 6H). Co-immunoprecipitation experiments found that an anti-PKD2 antibody could pull down PLN, and that an anti-PLN antibody could reciprocally pull down PKD2 proteins (Fig. 6I), suggesting a physical interaction between PKD2 and PLN.

To further validate that PLN phosphorylation can regulate SERCA-mediated Ca^2+^ activity, we utilized TAT-PLN-short, a TAT-conjugated short PLN fragment that contains both phosphorylation sites Ser-16 and Thr-17 but cannot bind to/regulate SERCA due to the lack of its C-terminal transmembrane domain and certain SERCA binding sites [33, 34]. TAT-PLN-short provides an excessive amount of membrane-permeant exogenous PLN phosphorylation substrates, which compete with endogenous PLN for a limited amount of endogenous protein kinases, causing a decreased phosphorylation of endogenous PLN, consequently reducing SERCA activity. Indeed, treatment of H7-CMs with TAT-PLN-short markedly reduced the amplitude and prolonged the decay kinetics of caffeine-induced Ca^2+^ transient (Fig. S8), indicative of a decreased SERCA activity.

### Relationships among SERCA activity, Ca^2+^ abnormality and ER stress

A selective SERCA inhibitor, thapsigargin, was used to explore the relationships among SERCA activity, Ca^2+^ abnormality, and ER stress. Thapsigargin treatment abolished the caffeine-induced cytosolic Ca^2+^ transient in H7-CMs (Fig. S9A and B), indicative of depleted SR Ca^2+^ stores after thapsigargin. Thapsigargin also elevated the ER stress level in H7-CMs, as indicated by increased expression levels of ER stress markers GPR78, CHOP, and ATF4 (Fig. S9C).

We also used the Crispr/Cas-based method to knockout the PKD2 gene in another hESC line H1. In agreement with the results from shRNA-based PKD2 knockdown in H7-CMs, Crispr/Cas-based PKD2 homozygous gene knockout impaired the SERCA-mediated Ca^2+^ signaling (Fig. S10A and B), elevated ER stress level (Fig. S10C), and decreased the expression levels of SERCA and phosphorylated PLN (Fig. S10D).

### SERCA activator CDN1163 could partially rescue the contractile deficiency of PKD2 knockout 3D hvCTS

**Fig. 7:**
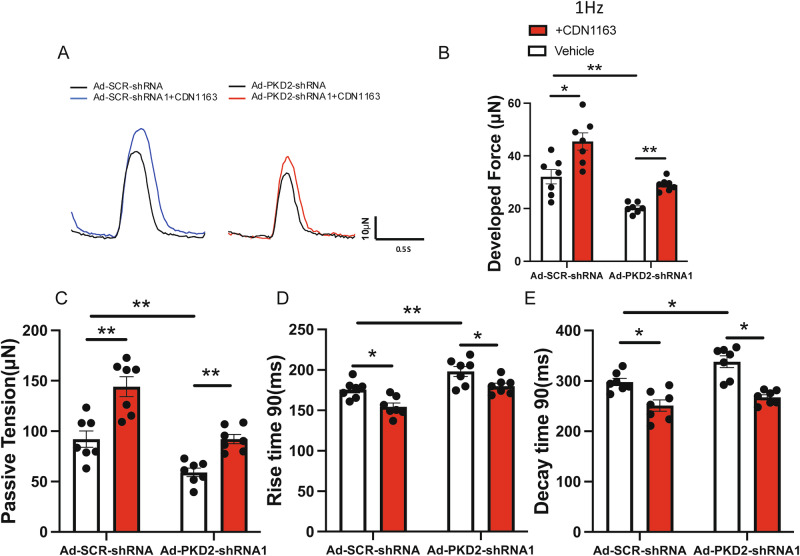
SERCA activator CDN1163 partially rescued the PKD2 deficiency-associated contractile deficiency. **A** Representative contractile traces of H7-CMs-hvCTS under 1 Hz electrical pacing before (black trace) and after (red or blue trace) 1 hr treatment with 1 μM CDN1163. The hvCTS samples with PKD2 knockdown are on the right, whereas those without PKD2 knockdown are on the left. Data summary showing that CDN1163 treatment improved the contractile function in hvCTS samples with or without PKD2 knockdown, regarding developed force (**B**), passive tension (**C**), rise time 90 (**D**), and decay time 90 (**E**). Mean ± SEM. *n* = 7. *P < 0.05; **P < 0.01; ***P < 0.001.

CDN1163 is a small molecular allosteric activator of SERCA2 that can increase Ca^2+^ reuptake into SR [35]. CDN1163 treatment increased the developed force and passive tension in both the PKD2 knockdown group and control group (Fig. 7A–C). CDN1163 treatment also decreased the rise time 90 and decay time 90 in both groups (Fig. 7D and E), indicative of better contraction velocity and relaxation velocity after CDN1163 treatment.

### PKD2 knockdown did not alter the features of cardiac microstructure

H7-CM-derived hvCTS was embedded into Tissue Tek OCT (Sakura, CA, USA), then cut into 10 mm-thick serial sections. Hematoxylin–eosin (H&E) staining was performed to observe the heterogeneous microstructural organization of hvCTS. The results showed no obvious microstructural differences between the PKD2 knockdown group and the control group (Fig. S11A). H7-CM-derived hvCTS sections were also stained for sarcomere protein α-actinin, which again showed no obvious differences between the PKD2 knockdown group and the control group (Fig. S11B).

## Discussion

Compared to 2D cultured cardiomyocytes, 3D bioengineered cardiac tissues can better mimic the structure and function of the native human heart [21–23, 36]. The 3D microenvironment enhances the maturation of hESC-CMs and promotes cardiomyocyte alignment for large force generation [21–23]. 3D bioengineered cardiac tissues also allow the measurement of contractile properties of properly aligned cardiomyocytes, which cannot be done with 2D cultured cardiomyocytes [21–23]. Previously, strip-like bioengineered cardiac tissues generated from hESC-CMs have been used as disease models to study the contractile defects of dilated cardiomyopathy [37] and hypertrophic cardiomyopathy [38]. In the present study, we bioengineered 3D hvCTS [21, 22] to recapitulate PKD2 deficiency-associated cardiac contractile defects. Our results clearly showed that PKD2 knockdown reduced the developed force, basal tension, and total tension of hvCTS. Furthermore, PKD2 knockdown slowed down the rise time kinetics and decay time kinetics of developed force. These data demonstrated that PKD2 knockdown reduced the contraction force and slowed down the contraction and relaxation velocities, all of which support the notion that PKD2 knockdown impairs the contractile properties of hvCTS. In the Ca^2+^ study, we also found that PKD2 knockdown reduced the magnitude of Ca^2+^ transients and slowed down the decay kinetics of Ca^2+^ transients in hESC-CMs, which could be the underlying reasons for the contractile deficiency [39].

Regarding the molecular mechanisms by which PKD2 deficiency could induce cardiomyopathies, previous studies from us and others reported that PKD2 deficiency may impair autophagy to induce cardiomyopathy [15, 16]. In the present study, we explored whether PKD2 deficiency could elevate ER stress as another culprit for cardiomyopathy. Several lines of evidence supported this notion. (1) PKD2 knockdown markedly reduced the magnitude of electrical pacing-induced cytosolic Ca^2+^ transients and SR Ca^2+^ release, suggestive of a reduced SR Ca^2+^ content in PKD2 knockdown cells. (2) A reduced SR Ca^2+^ content is expected to elevate ER stress [16], which was validated by our thapsigargin experiments (Fig. S9). Importantly, PKD2 knockdown indeed increased the expression levels of ER stress markers in both 2D hESC-CMs and 3D hvCTS. (3) Flow cytometry studies and TUNEL assay confirmed that PKD2 knockdown promoted apoptosis and/or necrosis of cardiomyocytes. (4) Small molecular chaperones 4-PBA and TUDCA, which are capable of relieving ER stress, partially restored the contractile deficiency in PKD2 knockdown hvCTS. Together, these results support that PKD2 knockdown dysregulates Ca^2+^ signaling to elevate ER stress, causing contractile dysfunction.

As to the question of how PKD2 deficiency could dysregulate Ca^2+^ signaling in cardiomyocytes, we and others have previously reported that PKD2 deficiency may disrupt its interaction with RyR2 to dysregulate Ca^2+^ signaling [5, 16]. Several studies also investigated the possible regulatory action of PKD2 on SERCA expression in mouse cardiomyocytes, but generated inconsistent results [5, 13, 17]. The discrepancy could be at least partly attributed to age-dependent variations [12]. In the present study, we focused on the effect of PKD2 deficiency on SERCA using hESC-CMs. The results showed that (1) PKD2 knockdown reduced the expression level of SERCA, as indicated by RT-qPCRs, western blots and cellular immunostaining; (2) PKD2 knockdown decreased the phosphorylated PLN, as demonstrated by western blots and cellular immunostaining. A decreased phosphorylation of PLN is expected to reduce the SERCA activity [32], which was validated by our TAT-PLN-short competition experiments. (3) PKD2 knockdown reduced the SERCA activity, as indicated by a decreased magnitude and a prolonged decay kinetics of cytosolic Ca^2+^ transients in PKD2 knockdown hESC-CMs [8].

Note that in 3D hvCTS, PKD2 knockdown-induced apoptosis was relatively minor (~2% of increase after PKD2 knockdown) (Fig. 3G and I). This was partly because we only used the best quality cardiomyocytes with vigorous spontaneous beating to construct 3D hvCTS, and partly because 3D hvCTS were maintained in good nutrition, and only the hvCTS with good basal tension were selected for contraction studies. Therefore, the contractile deficiency in 3D hvCTS could mostly be attributed to other factors, including (1) SERCA-related Ca^2+^ abnormality and (2) ER stress-induced contraction dysfunction and cardiomyopathy (not to the degree of cell death).

Our current hypothesis about the alteration of SERCA activity and ER stress in PKD2 deficient-cardiomyocytes have intriguing implications for cardiomyopathy treatment in ADPKD patients. Currently, there are no targeted treatment strategies for cardiomyopathies in ADPKD patients. In this study, we found that small molecular chaperones 4-PBA/TUDCA and SERCA activator CDN1163 could partially restore the contractile deficiency in PKD2 knockdown hvCTS. Note that SERCA activators have been previously used to treat heart failure patients [40], whereas small molecular chaperones 4-PBA and TUDCA are approved for the treatment of urea cycle disorders and/or biliary cirrhosis [41, 42]. Based on our studies, it is tempting to repurpose these agents to rescue PKD2 deficiency-associated cardiomyopathies in ADPKD patients. Note that in our study, 4-PBA, TUDCA, and CDN1163 also improved the contractile function in the control hvCTS without PKD2 knockdown. This was not surprising because other factors, such as lack of in vivo chemokines/cytokines and lack of interacting endothelial cells, may also elevate ER stress in 3D hvCTS, and furthermore, SERCA activator is known to improve cardiac function in normal rodents [43]. Interestingly, we found that the percentage of contractile improvement to 4-PBA/TUDCA was greater in PKD-deficient hvCTS than in control hvCTS. Together, our study suggests that small molecular chaperones and SERCA activators are beneficial in improving cardiac function in ADPKD patients, partly via relieving PKD2 deficiency-induced ER stress and SERCA abnormalities.

One limitation of using hESC-CMs is that these cells are relatively immature when compared to native human adult cardiomyocytes [44]. hESC-CMs display much smaller amplitude and slower kinetics in Ca^2+^ transients, show a lack of T-tubule structure, and have a more depolarized resting membrane potential than those of adult cardiomyocytes, all of which resemble the properties of 16-18 weeks fetal ventricular cardiomyocytes rather than adult cardiomyocytes [45, 46]. In this regard, we have now used 3D hvCTS to partly overcome this shortcoming. 3D hvCTS contains a mixture of hESC-CMs, human fetal fibroblasts, and an appropriate matrix that can provide a better niche to promote maturation of hESC-CMs. Indeed, it is known that the 3D hvCTS display improved alignment and electrical conduction with a significantly increased action potential velocity and contractile force than 2D hESC-CMs [46]. A more related question is whether hESC-CMs and hvCTS are appropriate disease models for cardiomyopathies of ADPKD patients. Regarding this, although renal defects in ADPKD are usually manifested in adults [1], animal studies showed that PKD2 deficiency-associated cardiac defects often occur in infants and embryonic stages [4, 5, 14]. Furthermore, cardiac abnormalities in ADPKD patients could occur at an early young age, sometimes in infants [3, 47–49]. Therefore, we believe that 3D hvCTS are suitable disease models for cardiomyopathies in young ADPKD patients, especially infant patients.

## Conclusions

With the use of hvCTS, we demonstrated that PKD2 deficiency decreased contractile force and reduced the contraction velocity and relaxation velocity. We further uncovered two novel mechanisms through which PKD2 deficiency causes cardiomyopathies, namely an elevated ER stress level and a decreased SERCA activity. The present study used 3D hvCTS and 2D hESC-CMs to recapitulate PKD2 deficiency-associated contractile defects. The results provide novel insights about PKD2 deficiency-associated cardiomyopathies in ADPKD patients.

## Supplementary information

Supplemental Materials

Western blot

## Data Availability

All data from this study are available from the corresponding author upon reasonable request.
