# Supplementary material for: Investigating PKD2 deficiency-associated cardiomyopathies using hESC-cardiomyocytes and bioengineered 3D ventricular cardiac tissue strips
Source: Cell Death Dis. 2026 Mar 25;17(1):368. doi: 10.1038/s41419-026-08639-8 (PMC13039962; doi:10.1038/s41419-026-08639-8)
Supplement: Supplementary file 1 — Supplemental Materials [file 41419_2026_8639_MOESM1_ESM.pdf]

Investigating PKD2 deficiency-associated cardiomyopathies using hESC-cardiomyocytes and bioengineered 3D ventricular cardiac tissue strips

Jingxuan Li[1,2,3], Wentao Peng[1,3], Maxwell Kwok[1], Huanyu Ding[1,3], Duan Zhuo1[1,3,4], Bimal Gurung[5], Ishan Raj Lakhani[1,3], Hongyan Yu[1,3], Ellen N Poon[1]*, Ronald A Li[5,6], Xiaoqiang Yao[1,3]*

[1]School of Biomedical Sciences, Faculty of Medicine, The Chinese University of Hong Kong, Institute of Hematology & Blood Diseases Hospital, Chinese Academy of Medical Sciences & Peking Union Medical College, Hong Kong, China.

[2]State Key Laboratory of Experimental Hematology, National Clinical Research Center for Blood Diseases, Haihe Laboratory of Cell Ecosystem, Tianjin 300020, China.

[3]Heart and Vascular Institute and Li Ka Shing Institute of Health Science, Faculty of Medicine, The Chinese University of Hong Kong, Hong Kong, China

[4]Southern University of Science and Technology Yantian Hospital, Shenzhen, China

[5]Novoheart, Boston, MA 02210, USA

[6]Medera Biopharm, Boston, MA 02210, USA

*Corresponding authors:
Xiaoqiang Yao, Ph.D.
School of Biomedical Sciences,
The Chinese University of Hong Kong,
Shatin, Hong Kong SAR, PRC China.
Phone: 852-39436877 ; Fax: 852-26035022
Email: yao2068@cuhk.edu.hk

Ellen N Poon, Ph.D.
School of Biomedical Sciences,
The Chinese University of Hong Kong,
Shatin, Hong Kong SAR, PRC China.
Phone: 852-39435750
Email: ellen.poon@cuhk.edu.hk

This PDF file includes: Figures S1-S11

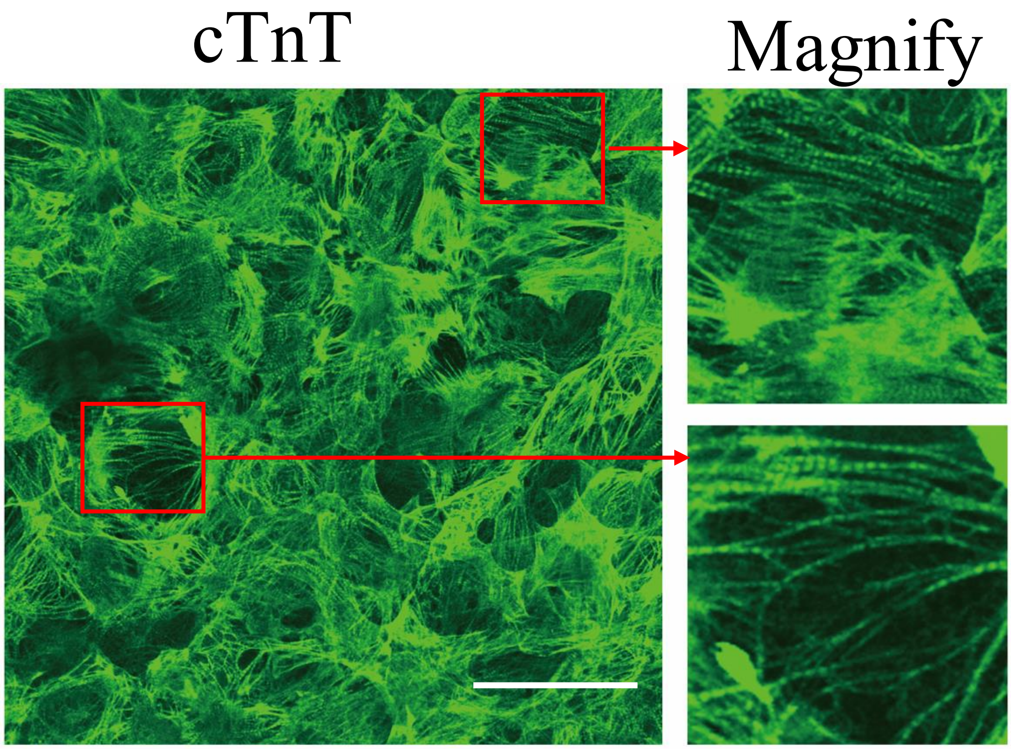

Figure S1. Sarcomeric organization of differentiated H7-CMs. Shown are confocal microscopy images of H7-CMs labelled with cardiac troponin T (Green), illustrating cardiomyocytes with aligned myofibrils and organized sarcomeres. Scale bar = 20 μm.

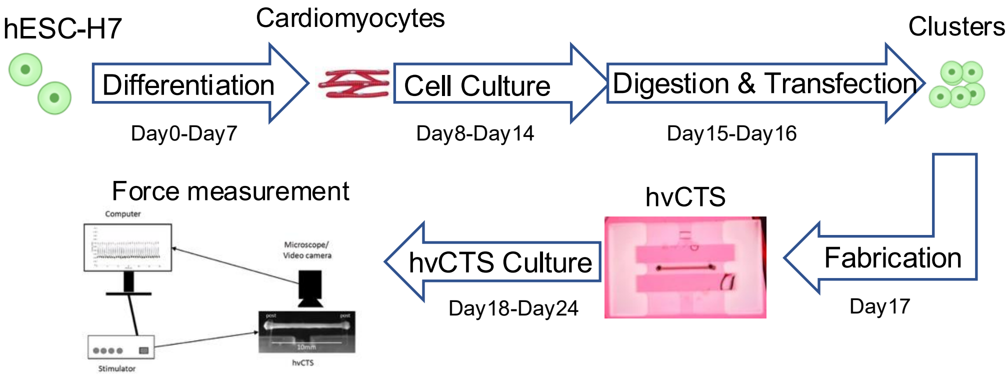

Figure S2. Schematic diagram of hvCTS fabrication and contractile force measurement. hESC (H7 or HES2) were differentiated into cardiomyocytes, then treated with or without adenoviral/lentiviral-shRNA and waited for 48 hrs (day15 - day 16) to form clusters. Clusters were fabricated into hvCTS, followed by contractile force measurement.

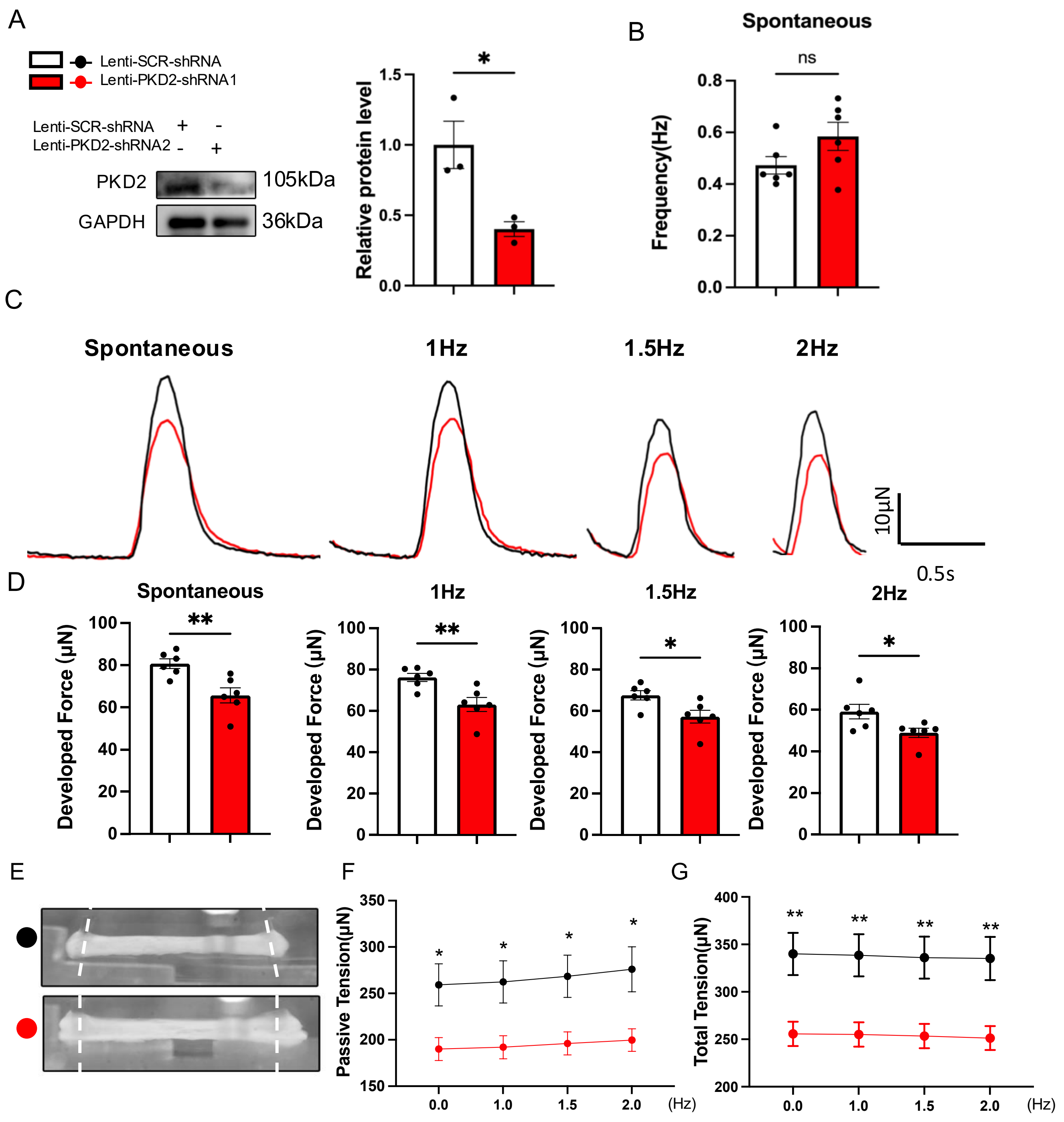

Figure S3: PKD2 knockdown reduced the contractile force of spontaneous and pacing-inducing contraction in HES2-CMs-derived 3D hvCTS. (A) Effectiveness of Lenti-PKD2-shRNA2 in knocking-down the expression of PKD2 at protein level (n = 3). Shown are representative immunoblot images (left) and summary data normalized to GAPDH (right). **(B)** Spontaneous frequency of hvCTS with or without PKD2 knockdown. (**C**) Representative contractile traces of hvCTS with or without PKD2 knockdown at different pacing frequency. (**D-H**) Data summary showing that PKD2 knockdown with Lenti-PKD2-shRNA2 reduced the developed force (D), passive tension (E-G) and total tension (G) at different pacing frequency. **E** illustrated the real images of resting hvCTS with (lower image) or without (upper image) PKD2 knockdown. Passive tension (or hvCTS shortening) was obvious in the upper panel, but not obvious in the lower panel. n = 6. Mean ± SEM. *, P< 0.05; **, P< 0.01; by Student *t* test in A, B, or two-way ANOVA in F, G.

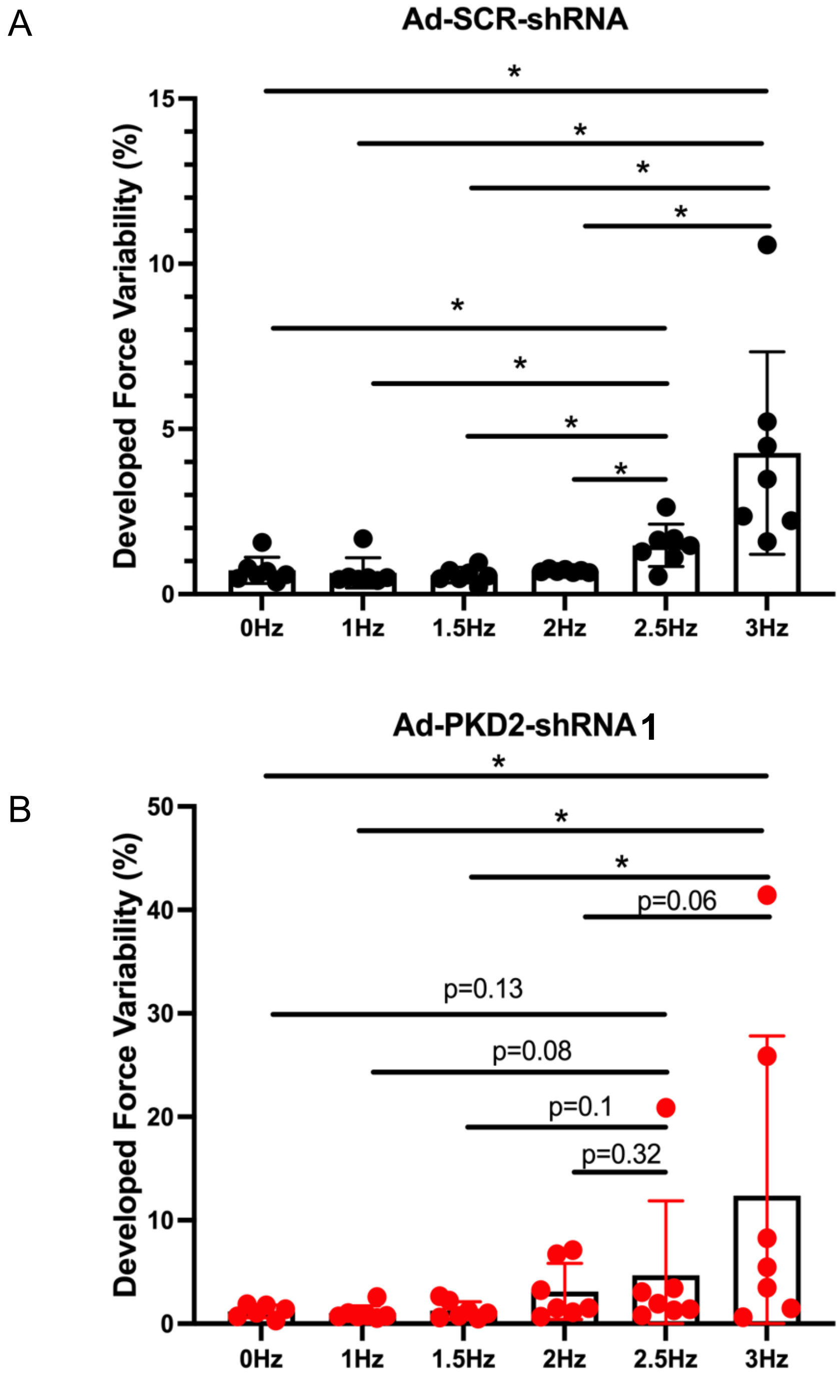

Figure S4. Variability in developed force measurement for hvCTS became excessive when electric pacing frequency reached 2.5 Hz and 3 Hz. Shown are summary data about the variability of developed force in % at different electrical pacing frequency. (A) H7-CMs-derived hvCTS transduced with Ad-SCR-shRNA1. n=7. (B) H7-CMs-derived hvCTS transduced with Ad-PKD2-shRNA1. n = 7. Mean ± SEM. *, $P < 0.05$; by one-way ANOVA.

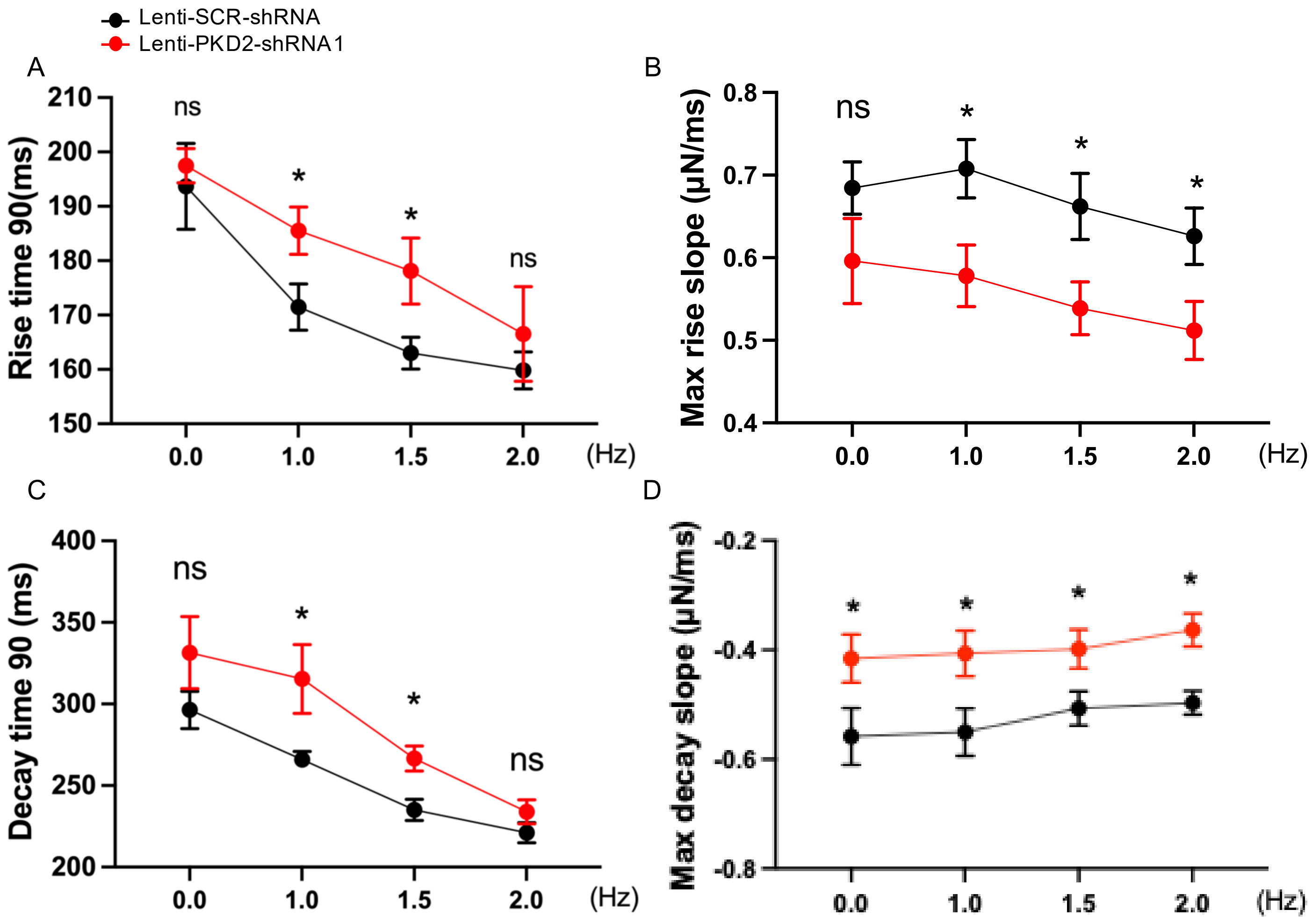

Figure S5. PKD2 knockdown slowed down the rise kinetics and decay kinetics of developed force in HES2-CMs-derived 3D hvCTS. Data summary showing that PKD2 knockdown with Lenti-PKD2-shRNA2 prolonged the rise time 90 (A) and decay time 90 (C), and decreased the maximal rise slope (B) and maximal decay slope (D) at different pacing frequency in HES2-CMs-derived 3D hvCTS. n = 6. Mean ± SEM. *, P< 0.05; **, P< 0.01; by two-way ANOVA.

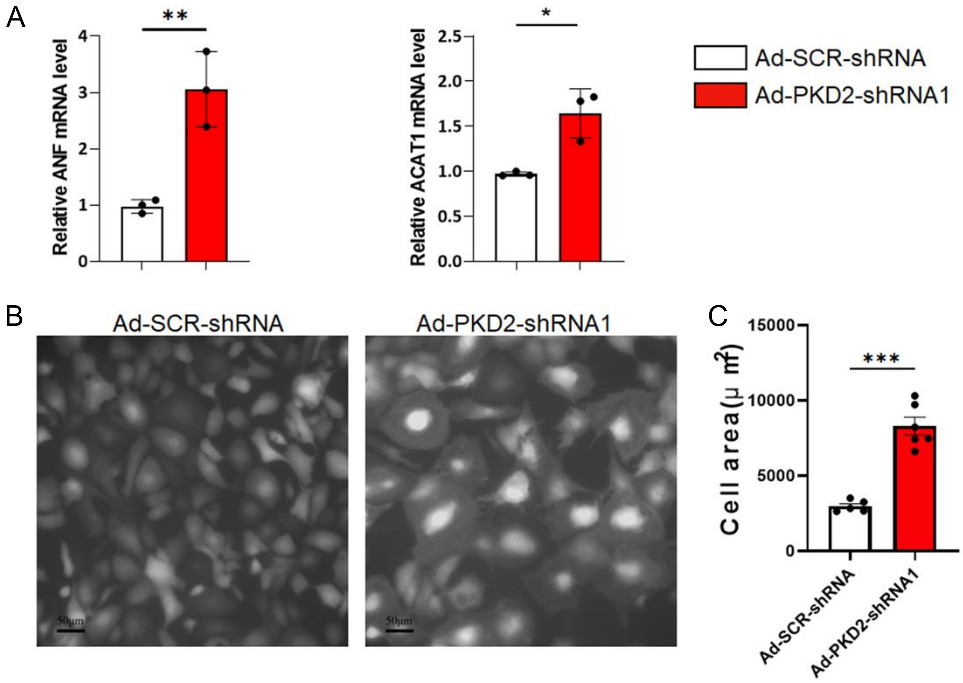

Figure S6. PKD2 knockdown induced cardiomyocyte hypertrophy. A). PKD2 knockdown increased the expression of ANF and ACAT1 mRNAs by RT-qPCR in H7-CMs. n = 3. **B).** Representative image of H7-CMs transfected with Ad-SCR-shRNA or Ad-PKD2-shRNA1. **C).** Quantification of cell area as in B. n = 5-6. Mean ± SEM. *, p<.05; **, p<.01; ***, p<.001.

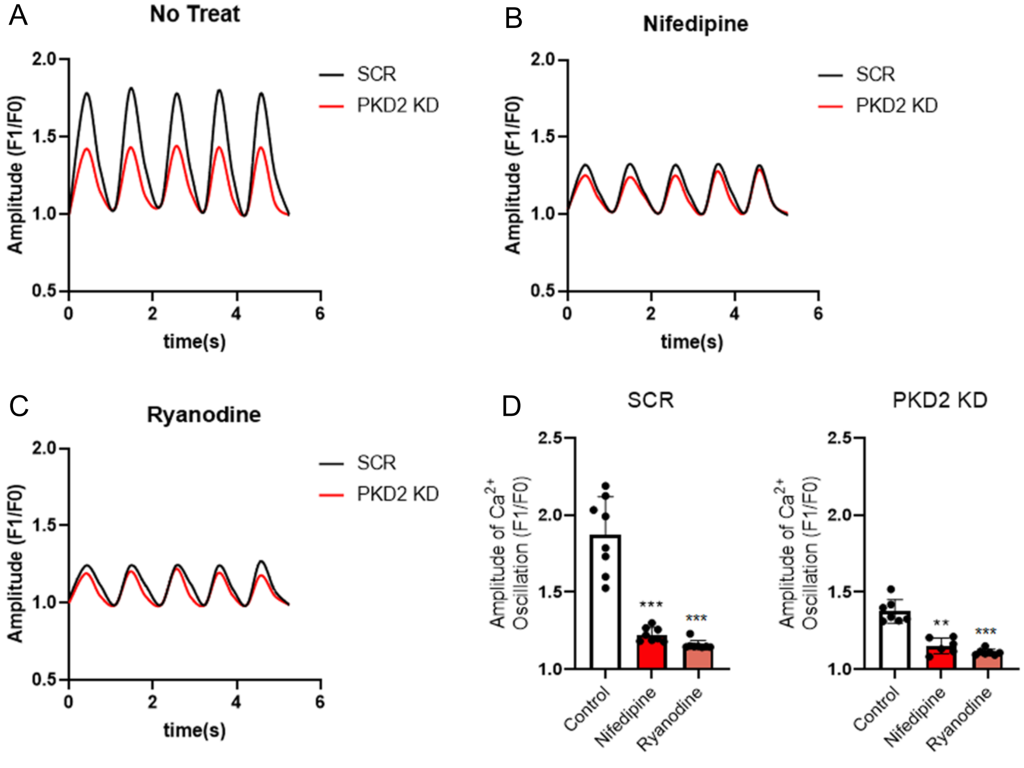

Figure S7. Involvement of L-type Ca^{2+} channels and RyR2 in electrical pacing-induced cytosolic Ca^{2+} oscillations in normal and PKD2 knockdown H7-CMs. Shown are representative traces (**A-C**) and quantification (**D**) of 1 Hz electrical pacing-induced cytosolic Ca^{2+} oscillations in H7-CMs. The cells were loaded with a fluorescent Ca^{2+} dye Calbryte 590 for 1 hr. The cells were treated with or without 10 µM ryanodine for 30 min or 5 µM nifedipine for 10 min in Tyrode's solution, then challenged with 1 Hz electrical pacing. Mean ± SEM; n = 6 - 8. *, p<.05. **, p<.01. ***, p<.001.

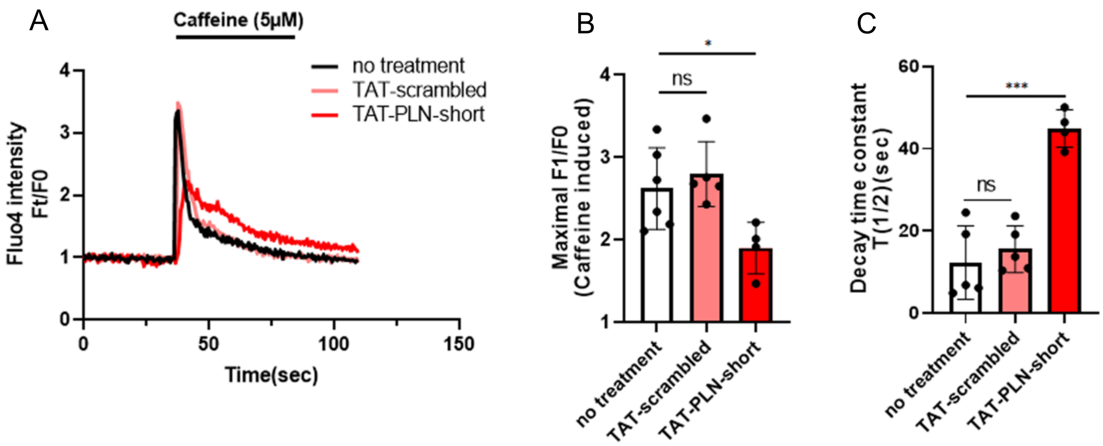

Figure S8. **Excessive supply of exogenous PLN phosphorylation substrates (TAT-PLN-short) reduced the SERCA activity.** H7-CMs were treated with or without 30 μM TAT-scrambled or TAT-PLN-short for 45 min before loading with a fluorescent Ca^{2+} dye Calbryte 590 for 1 hr. The cells were incubated in Ca^{2+}-free Tyrode's solution, then challenged with 5 μM caffeine. The reduced SERCA activity was indicated by decreased amplitude and prolonged decay time kinetics of caffeine-induced Ca^{2+} transients. (**A**). Representative trace of caffeine-induced SR Ca^{2+} release. (**B**). Quantification of maximal Ca^{2+} amplitude in response to 5 μM caffeine. (**C**). Quantification of decay time kinetics in response to 5 μM caffeine. Mean ± SEM. n ≥ 5. ns, not significant; *, p<.05; ***, p<.001.

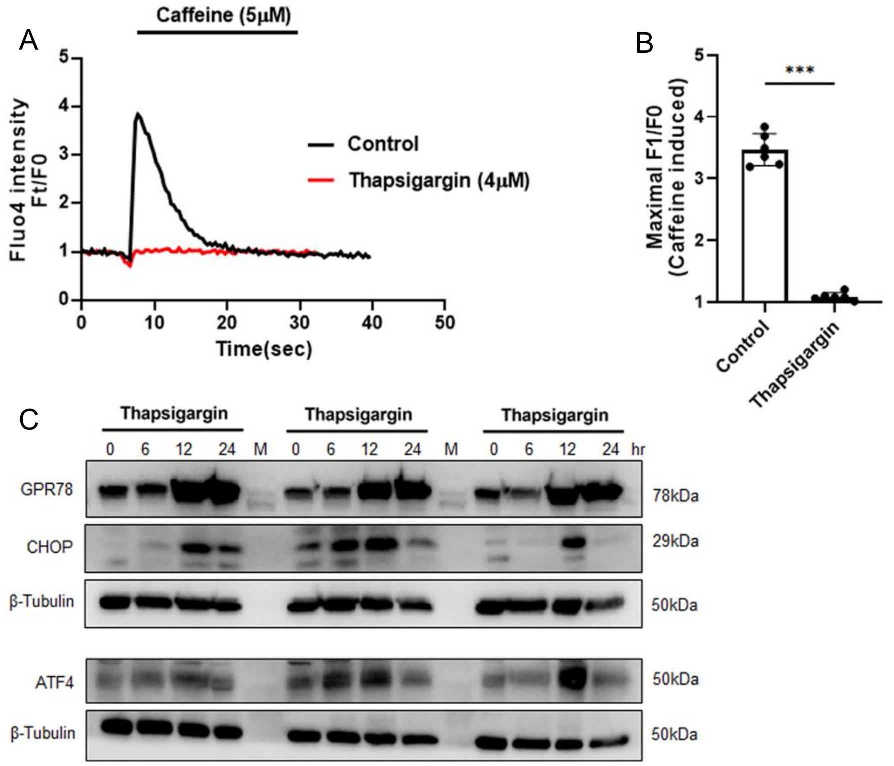

Figure S9. **Thapsigargin treatment abolished the caffeine-induced cytosolic Ca^{2+} transient and elevated the ER stress in H7-CMs.** (**A-B**) Representative trace (A) and quantification (B) of caffeine-induced cytosolic Ca^{2+} transient. The cells were pretreated with or without 4 μM thapsigargin for 10 min after loading with a fluorescent Ca^{2+} dye Calbryte 590 for 1 hr. The cells were incubated in Ca^{2+}-free Tyrode's solution, then challenged with 5 μM caffeine. Mean ± SEM; n = 6. ***, p<.001. (**C**) Representative immunoblots showing that thapsigargin treatment (100 nM for different time durations) increased the expression of ER stress markers including GRP78, ATF4 and CHOP. M, molecular marker lane. n = 3.

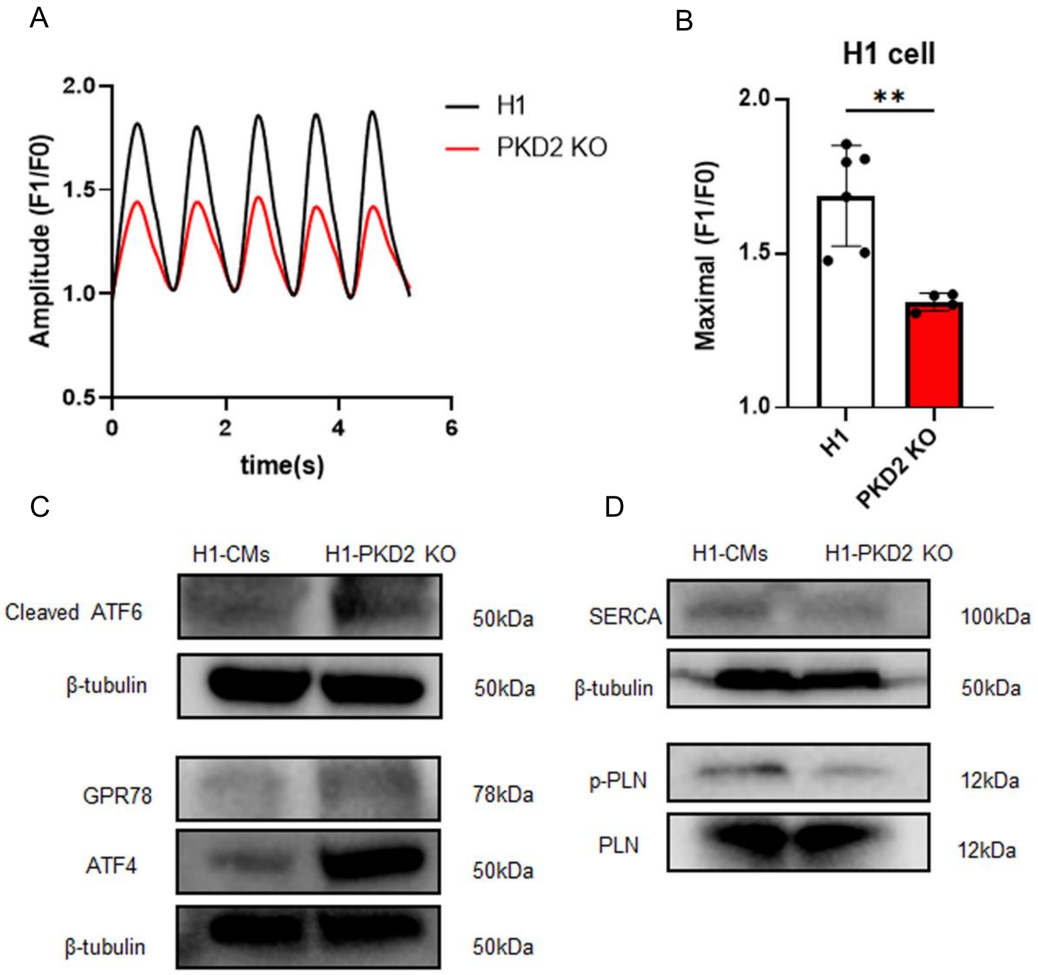

Figure S10. **Crispr/Cas9-based homozygous PKD2 gene knockout decreased the magnitude of cytosolic Ca^{2+} oscillations, elevated ER stress and decreased the expression of SERCA and phosphorylated PLN. (A-B)** PKD2 gene knockout reduced the magnitude of cytosolic Ca^{2+} oscillations in H1-CMs under 1 Hz electrical pacing. Shown are representative traces of cytosolic Ca^{2+} oscillations (A) and data summary of maximal amplitude value (B). Mean ± SEM; n = 4-6. **p<0.01. (**C)** Representative immunoblots showing that PKD2 gene knockout increased the expression of ER stress markers including cleaved ATF6, GPR78 and ATF4. n =3. **D)** Representative immunoblots showing that PKD2 gene knockout decreased the expression of SERCA and phosphorylated PLN. n =3.

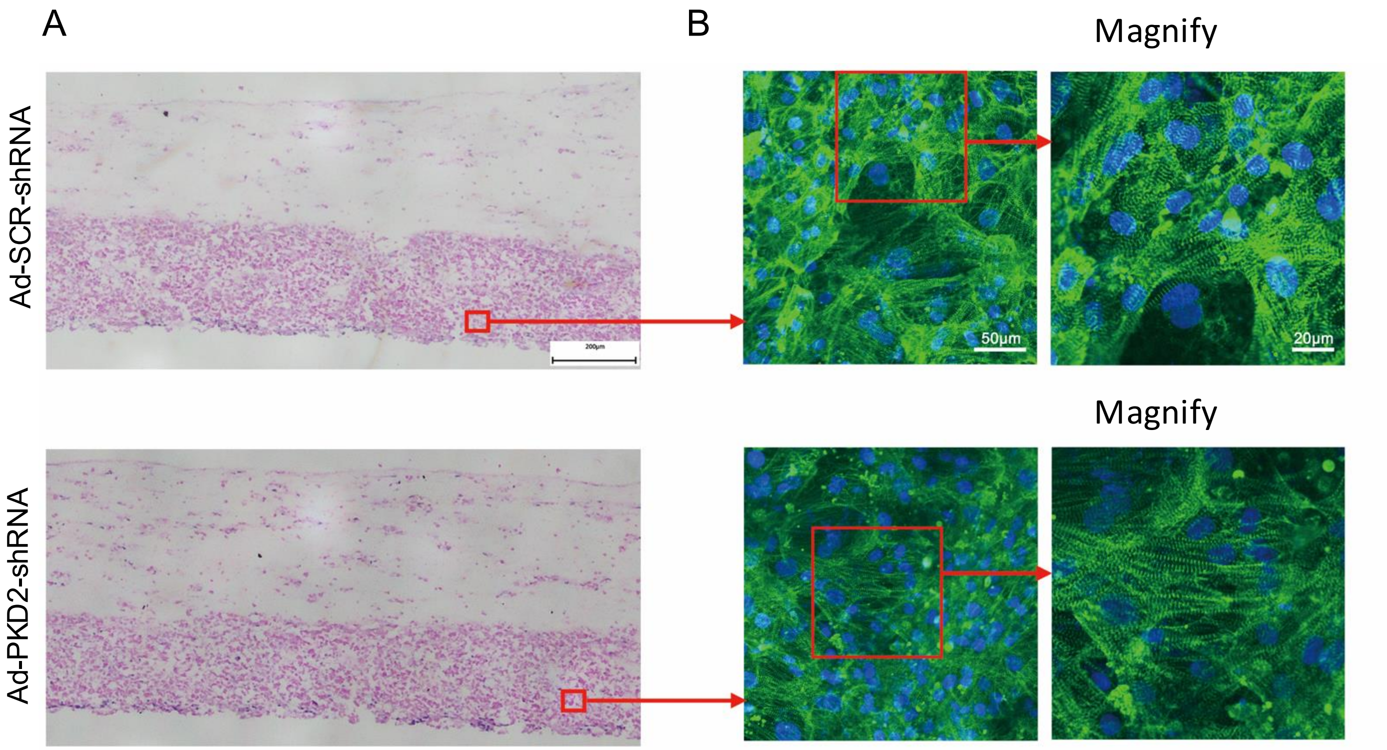

Figure S11. PKD2 knockdown did not alter the features of cardiac microstructure. Structural and molecular analysis of hvCTS. (**A**) H&E stain of hvCTS sections showed similar microstructural features of hvCTS with or without PKD2 knockdown. Note the cardiomyocytes were mostly distributed at the bottom side of the strips due to gravity. (**B**) Confocal microscopy pictures of hvCTS labelled with cardiomyocyte structural protein α-actinin (green) and DAPI (blue). The pictures were taken from cardiomyocytes-enriched regions (small red boxes in A). Clear sarcomere structures could be observed in magnified images on the right. No obvious structural differences could be observed between PKD2 knockdown group (Ad-PKD2-shRNA) and the control group (Ad-SCR-shRNA). Scale bar in A = 200 μm.
