# Supplementary material for: Investigating PKD2 deficiency-associated cardiomyopathies using hESC-cardiomyocytes and bioengineered 3D ventricular cardiac tissue strips
Source: Cell Death Dis. 2026 Mar 25;17(1):368. doi: 10.1038/s41419-026-08639-8 (PMC13039962; doi:10.1038/s41419-026-08639-8)

Figure 1C

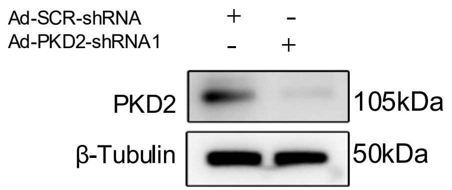

Original Blots related to Figure 1C and Figure 3A (2D samples in upper row and 3D samples in lower row)

Note that in our western Blot expts, we used scissors to cut the membranes into 2-3 pieces, each of which was used to detect different targeting proteins.

Figure 3A

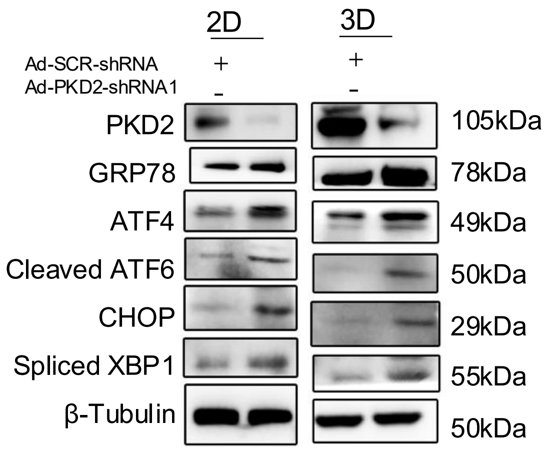

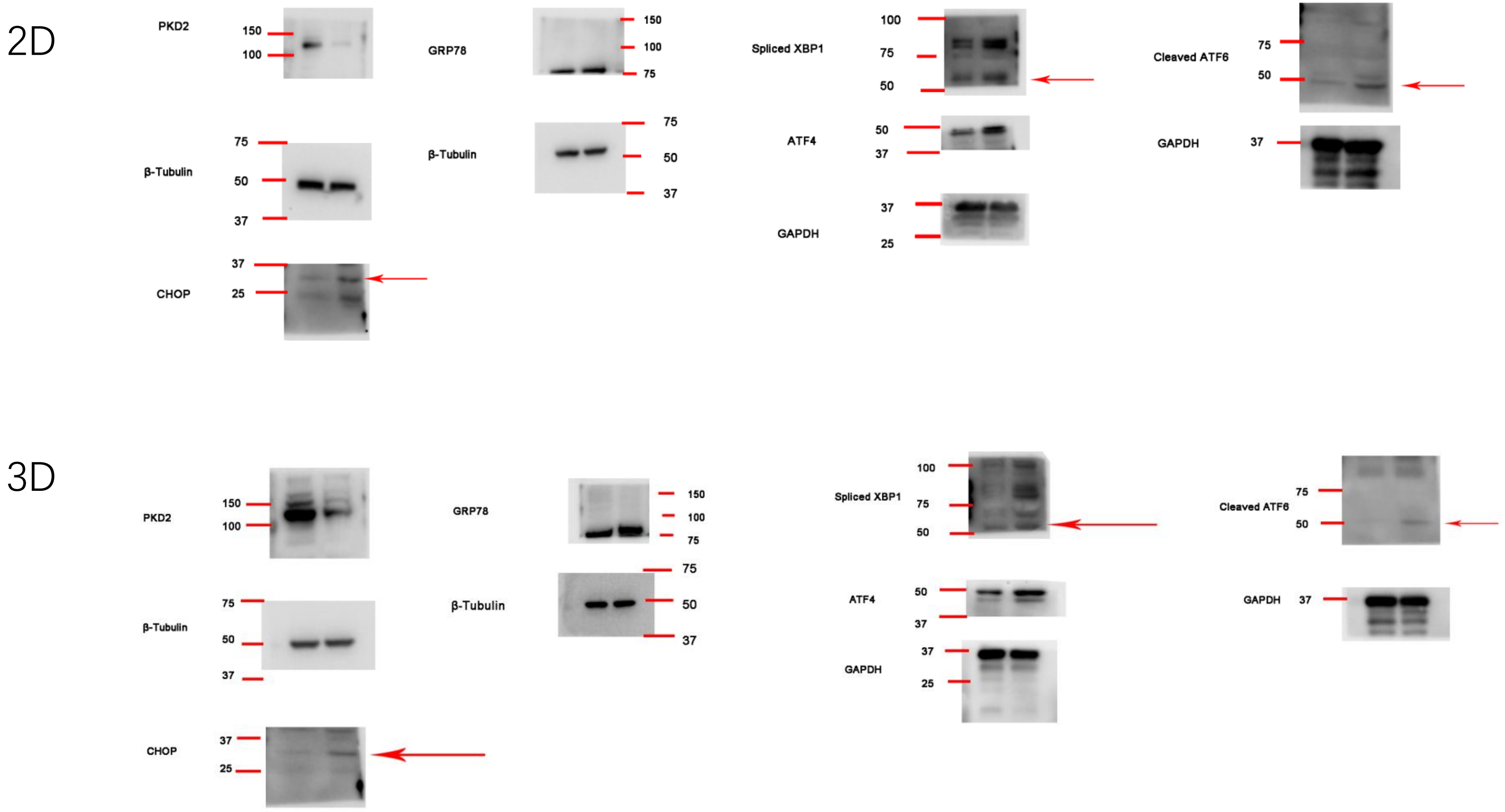

Figure 6B

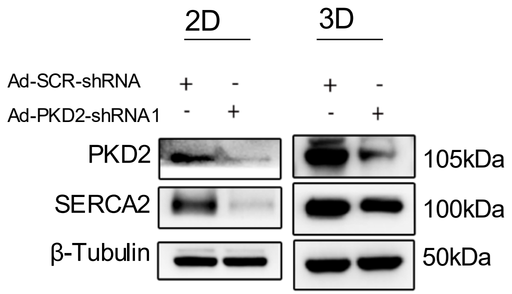

Original Blots related to Figure 6B

In our western Blot expts, we used scissors to cut the membranes into several pieces, each of which was used to detect different targeting proteins. Also note that because the MWs of PKD2 and SERCA proteins are very close, the same blotted membrane was used for detection of two proteins. After detection of the first protein, the membrane was striped of the first antibody, followed by incubation with another antibody then detection.

In addition, please note that the same membrane was used for Fig. 6B (3D data) and Chop detection in Fig. 3A (3D data, with full blot displayed on the leftmost bottom column).

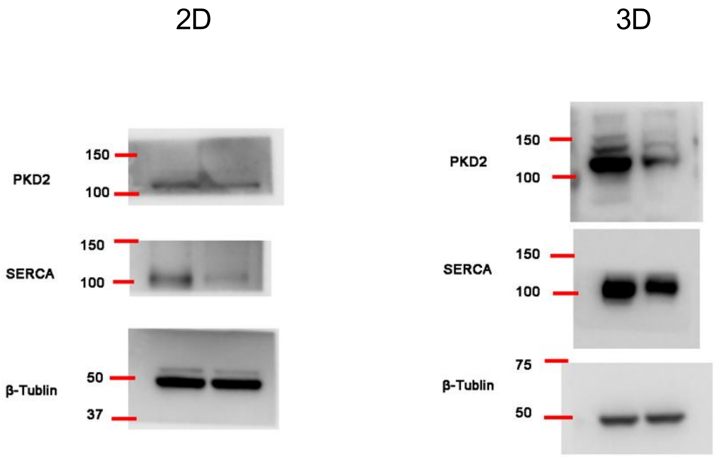

Figure 6F

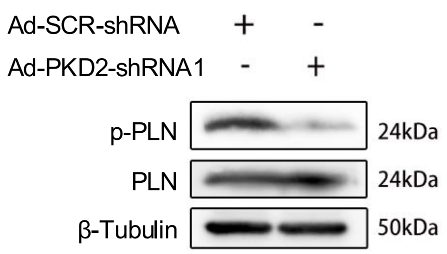

Original Blots related to Figure 6F

Note that in our western Blot expts, we used scissors to cut the membranes into 2-3 pieces, each of which was used to detect different targeting proteins.
Also note that because the p-PLN and PLN have similar MW, the same blotted membrane was used for detection of two proteins. After detection of the first protein, the membrane was striped of the first antibody, followed by incubation with another antibody then detection.

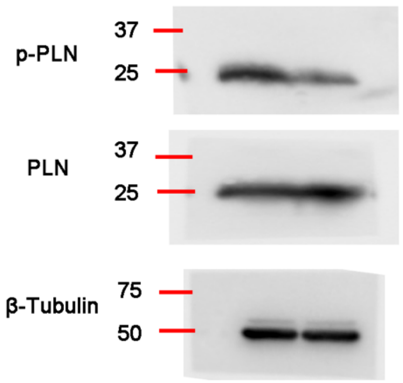

Figure 6I

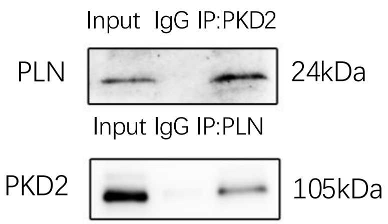

Original Blots related to Figure 6I

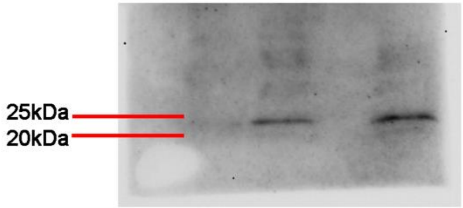

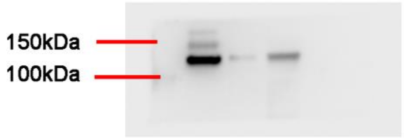

Figure S3A

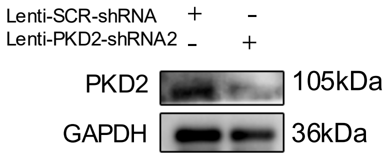

Original Blots related to Figure S3A

Note that in our western Blot expts, we used scissors to cut the membranes into 3 pieces, each of which was used to detect different targeting proteins.

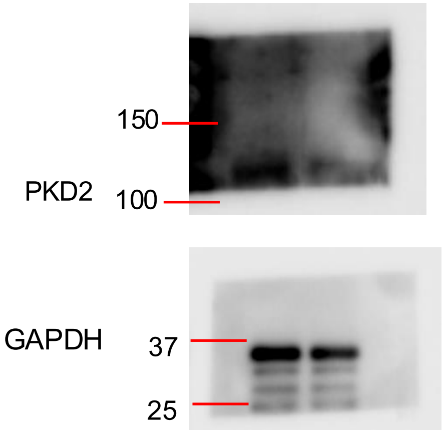

Figure S9C

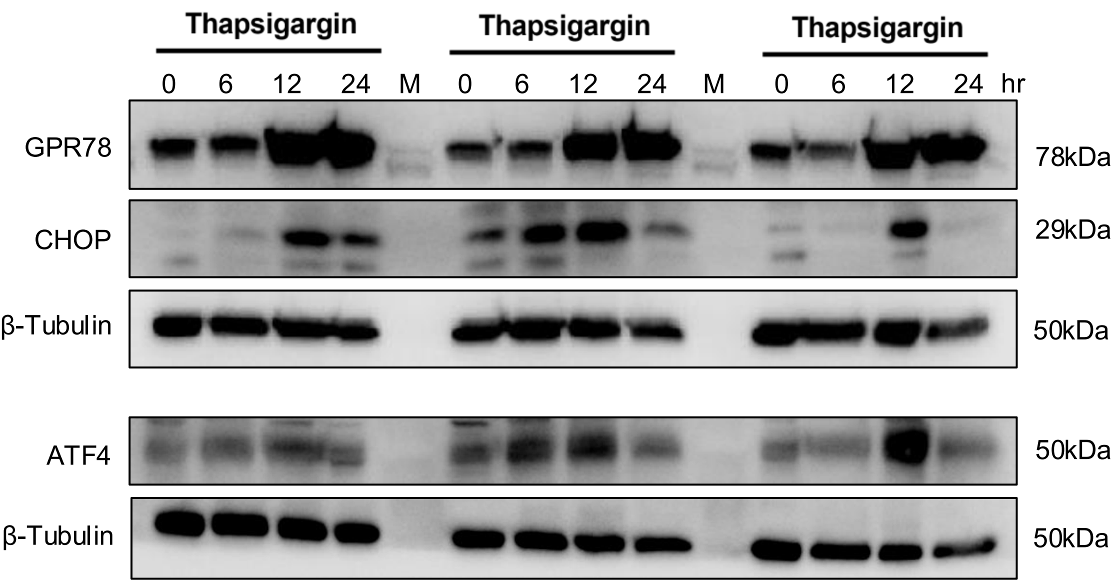

Original Blots related to Figure S9C

Note that in our western blot expts, we used scissors to cut the membranes into 2-3 pieces, each of which was used to detect different targeting proteins.

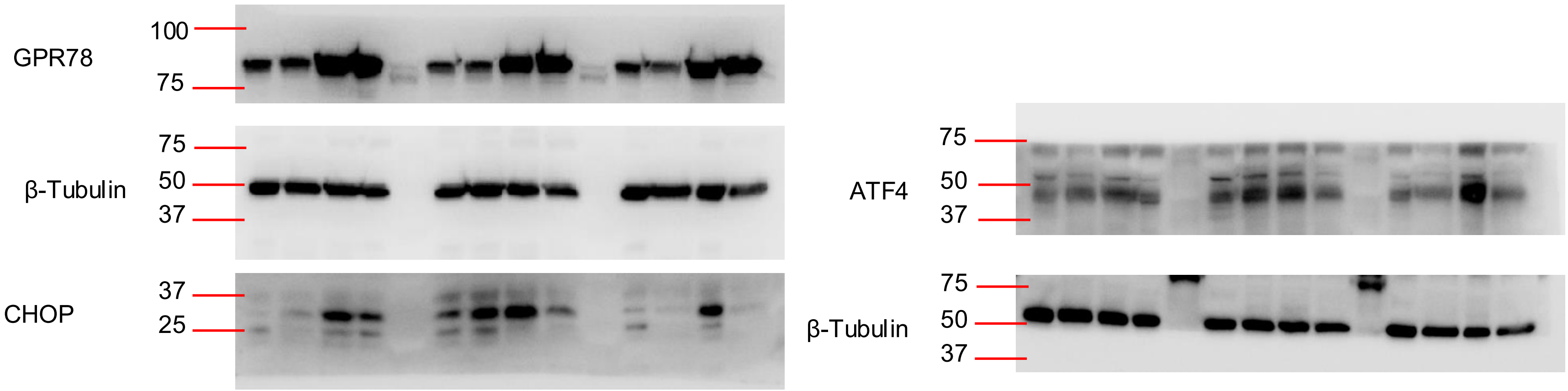

Figure S10C

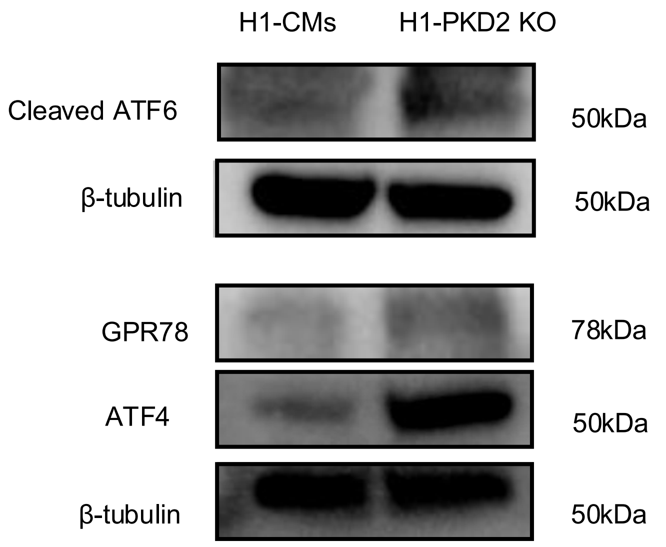

Figure S10D

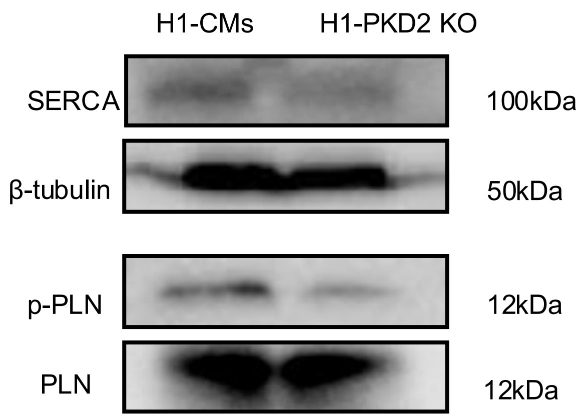

Original Blots related to Figure S10C and S10D

Note that in our western blot expts, we used scissors to cut the membranes into 2-3 pieces, each of which was used to detect different targeting proteins.

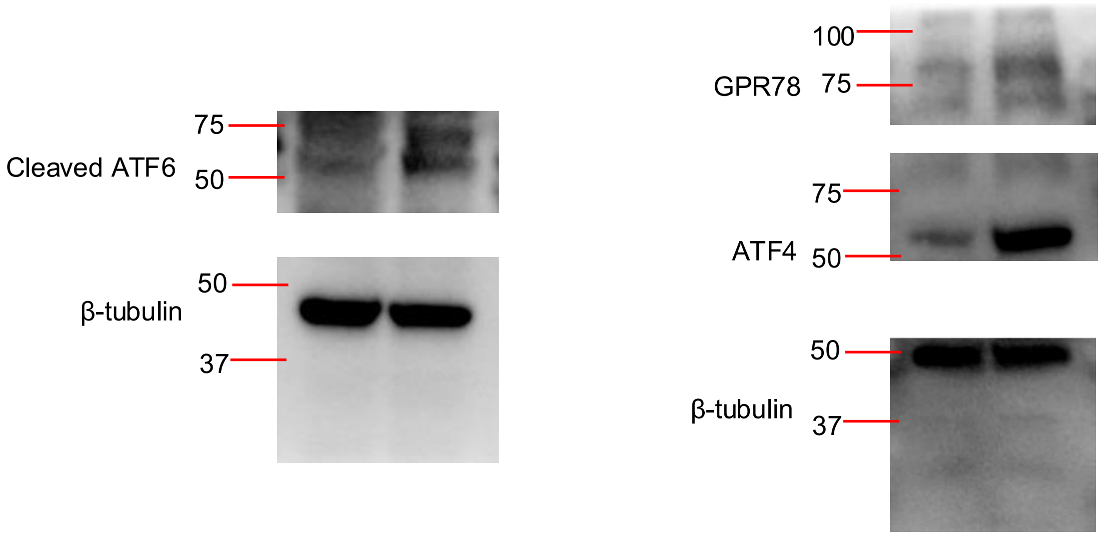

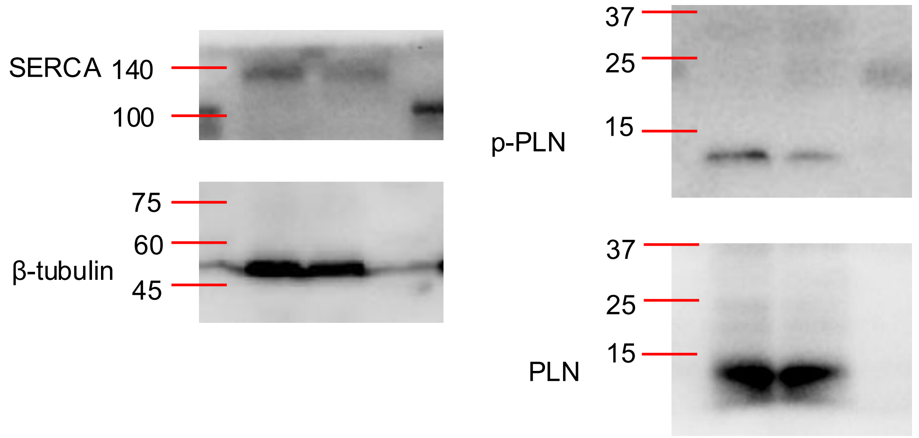

Supplement: Supplementary file 2 — Western blot [file 41419_2026_8639_MOESM2_ESM.pdf]
